# The Spatiotemporal Genetic Architecture of Seed Vigor in Upland Cotton

**DOI:** 10.1002/advs.76067

**Published:** 2026-06-12

**Authors:** Luyao Wang, Jie Dai, Ting Zhao, Shengjun Zhao, Hongyu Wu, Jiaying Pan, Yupeng Hao, Ke Nie, Li Yu, Yumeng Zhu, Xiangyi Zhao, Kai Huang, Mengke Zhang, Yongyan Zhao, Yaping Jiang, Siyuan Wang, Yiling Pan, Yubing Chen, Yilai Lin, Wanghong Shi, Yayuan Deng, Jin Han, Jie Li, Xuan Long, Junbin Wang, Zhiyuan Zhang, Zegang Han, Zhanfeng Si, Lei Ju, Jun Li, Tianzhen Zhang, Ji Zhou, Xueying Guan

**Affiliations:** ^1^ Cotton and Cash Crops Innovation Group Hainan Institute of Zhejiang University Yazhou Bay Science and Technology City Sanya Hainan China; ^2^ Engineering Research Centre of Cotton Ministry of Education / College of Agriculture Xinjiang Agricultural University Urumqi China; ^3^ Tropical Crops Genetic Resources Institute Chinese Academy of Tropical Agricultural Sciences/Key Laboratory of Crop Gene Resources and Germplasm Enhancement in Southern China Ministry of Agriculture Haikou China; ^4^ State Key Laboratory of Plant Trait Design CAS Center for Excellence in Molecular Plant Sciences (CEMPS) Shanghai Institute of Plant Physiology and Ecology (SIPPE) Chinese Academy of Sciences (CAS) Shanghai China; ^5^ Zhejiang Provincial Key Laboratory of Crop Genetic Resources The Advanced Seed Institute Plant Precision Breeding Academy College of Agriculture and Biotechnology Zhejiang University Hangzhou China; ^6^ Data Sciences Department Crop Science Centre National Institute of Agricultural Botany (NIAB) University of Cambridge Cambridge UK

**Keywords:** candidate gene, cotyledon, domestication, genetic architecture, genomics, heritability, pleiotropy, quantitative trait locus, radicle

## Abstract

Seed vigor underpins uniform crop establishment, but its dynamic genetics are understudied. Combining high‐resolution temporal phenotyping and genomics in upland cotton, we used the SeedRanger platform to record 17 image‐based traits every 30 min over 120 h, revealing stage‐specific heritability and identifying 541 seed‐vigor loci. These loci show extensive pleiotropy and temporal coordination, forming a genetic network that preserves developmental continuity; 8.9% overlap regions under domestication selection, indicating concurrent optimization with fiber yield. Functional validation of *FLA2*, a candidate gene underlying a dynamic QTL, implicates auxin‐mediated control of radicle elongation and cotyledon development. This temporal framework exposes dynamic genetic architecture and breeding targets for high‐vigor crops.

## Graphical abstract

Leveraging the semi‐automated SeedRanger platform, we profiled the germination kinetics of 356 cotton accessions at a 30‐min interval. This high‐throughput phenomic approach delineated a temporal genetic network comprising 541 stage‐specific loci. Crucially, functional validation identified FLA2 as a pivotal, auxin‐modulated regulator that orchestrates radicle elongation and cotyledon expansion, thereby conferring synchronized germination and superior seed vigor.

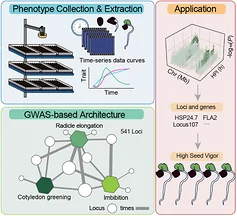

## 1 Introduction

Seed vigor, defined by the International Seed Testing Association (ISTA) as a core trait representing the sum of performance factors that determine acceptable seed germination activity and performance under diverse environmental conditions, offers significant advantages in describing germination rate, emergence rate, resistance to abiotic and biotic stresses, and storage capacity under varying conditions [1, 2]. It provides a comprehensive evaluation of seed performance, making its assessment highly significant. In modern agriculture, high‐vigor seeds significantly improve germination uniformity and seedling robustness [3], enhance early stress tolerance [4], reduce agricultural inputs, and lower the environmental footprint [5, 6], thereby directly supporting agricultural sustainability and food security. Since seed vigor is influenced by genetic background, seed maturity, and environmental conditions [7], establishing an accurate and reproducible phenotypic evaluation system is crucial for elucidating its underlying mechanisms and enabling breeding applications [8].

This trait of seed vigor encompasses multiple consecutive developmental stages, from imbibition and radicle emergence to chloroplast biogenesis [9], and integrates desirable characteristics such as rapid germination and seedling health under both optimal and suboptimal conditions [10]. Studying seed vigor at a fixed developmental stage is missing the forest for the trees, due to the temporal variability of its associated traits widely extended to the whole germination progress up to early seedling stage. As a composite trait affected by multiple factors [7], seed vigor necessitates the development of non‐destructive and scalable phenotyping tools to dynamically characterize its fitness and morphological factors throughout the germination process [8]. Although multi‐omics studies have attempted to address this challenge [11], the investigation are often limited by small sample sizes and static sampling strategies, providing only snapshots of germination at discrete time points [12, 13], thus failing to capture the biological dynamics of seed vigor. Despite advances in omics technologies, the capacity to analyze seed vigor dynamics at the population level remains limited [14].

Traditional research on seed vigor has primarily focused on traits such as seed dormancy, germination capacity, and chloroplast development. Since the 1990s, imaging technologies have been progressively applied to monitor seed growth, leading to platforms such as OSU SVIS [15], phenoSeeder [16], SAPL [17], SmartGrain [18] and Videometer Seed Lab [19]. However, current seed vigor assessment methods remain low‐throughput, labor‐intensive, and heavily reliant on manual operations, significantly limiting their scalability. In recent years, non‐destructive imaging technologies based on computer vision have offered a scalable and cost‐effective solution for seed phenotyping. For instance, tools like SeedGerm [14] enable detailed morphological quantification and individual seed tracking throughout germination.

**FIGURE 1:**
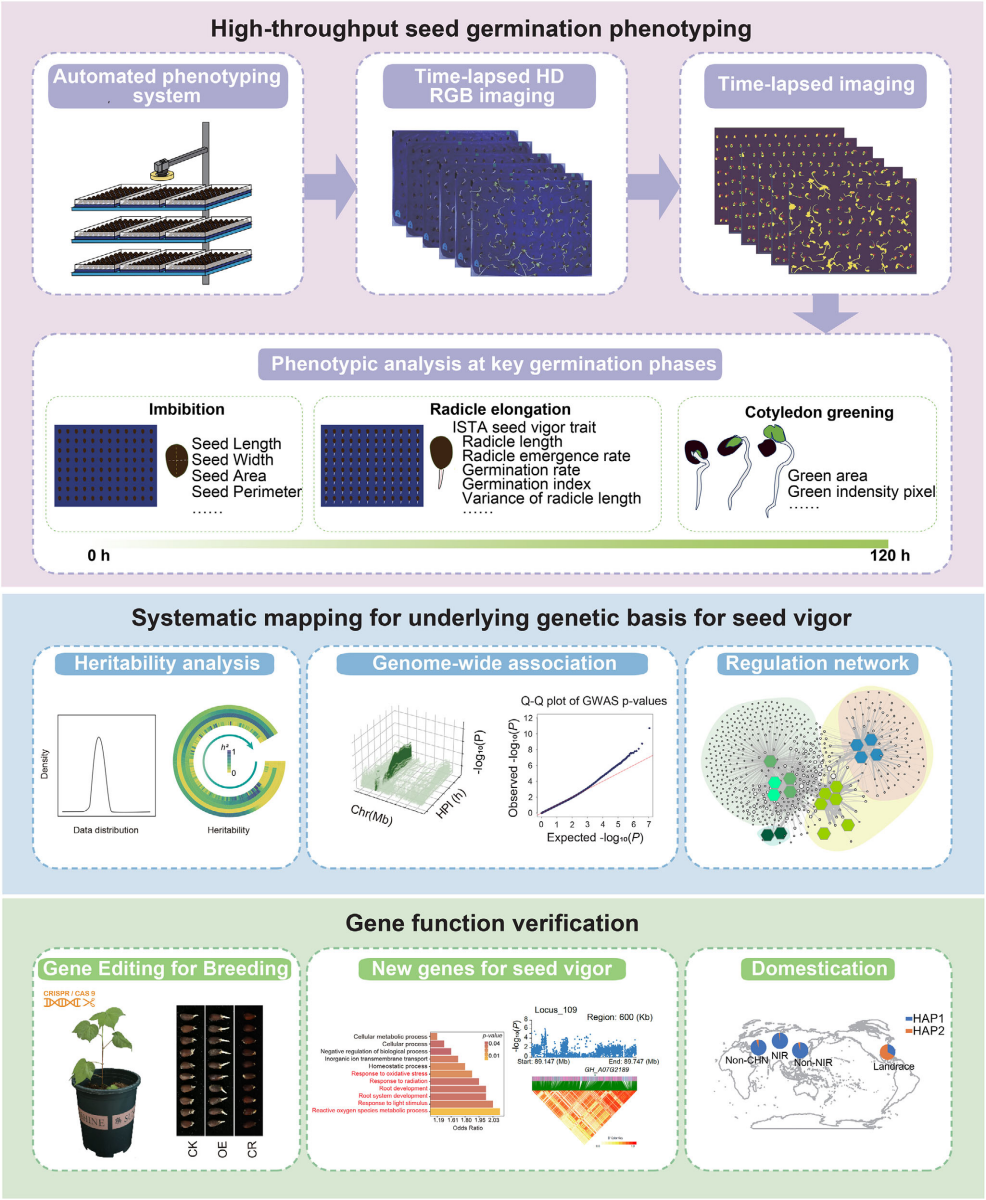
Integrated workflow for high‐throughput genetic dissection of cotton seed vigor. The automated seed vigor monitoring system, SeedRanger, was used to acquire RGB images of 104 seeds from each of 356 genetic accessions. Images were captured every 30 min from imbibition until 120 h post‐imbibition (HPI), covering germination through imbibition, radicle elongation and cotyledon greening. Quantitative image‐based traits (i‐Traits) were extracted using advanced computer vision algorithms. Temporal heritability analysis, genome‐wide association studies (GWAS), and regulatory network construction were performed to identify dynamic QTLS (dQTLs) underlying seed vigor. Putative candidate genes within the most significant QTL regions were functionally validated using CRISPR‐Cas9 gene editing, comparative GWAS, and analysis of seed domestication traits.

Cotton, with its typical taproot system and pronounced color contrast between the seed coat and radicle, provides an ideal system for using imaging technologies to dynamically monitor radicle elongation and study complex traits related to seed vigor. Compared to cultivated varieties, wild cotton germplasm often exhibits characteristics such as plant miniaturization, deep dormancy, and prolonged germination periods [20], reflecting the significant role of domestication in reducing seed dormancy and improving germination traits [21]. Although cotton possesses a high‐quality reference genome and comprehensive resequencing data covering landrace and cultivated species, the genomic selection mechanisms underlying seed yield and vigor‐related traits during domestication remain unclear. To dissect the genetic basis for cotton seed vigor, we developed SeedRanger, a novel germination monitoring system, to perform high‐temporal‐resolution imaging (every 30 min for 5 days) of the China Upland Cotton Cultivation Population 2 (CUCP2), systematically capturing the entire process of imbibition, germination, radicle elongation, and cotyledon greening. This work establishes a preliminary analytical framework for revealing the spatiotemporal dynamics of the genetic regulation of seed vigor (Figure 1).

## 2 Result

### 2.1 SeedRanger: A High‐Throughput Seed Vigor Phenotyping Platform

To systematically capture seed germination dynamics, we developed the SeedRanger semi‐automatic phenotyping platform (Video S1), which integrates a high‐resolution digital camera system for continuous RGB image acquisition at 30‐min intervals. The platform was installed in an environmentally controlled greenhouse with precise regulation of temperature and humidity. Its custom‐designed three‐axis translational mechanism enables movement along both horizontal and vertical axes of the cultivation rack. Upon reaching predetermined coordinates, the platform automatically retracts the germination chamber cover, and an articulated cantilever arm positions the imaging module directly above the target seeds with a positioning accuracy of 0.02 mm for top‐down‐view image capture. Each full imaging cycle is completed within 7 min. The platform incorporates a three‐tier rack system housing multiple germination chambers, with each tier capable of imaging nine discrete 30 cm × 40 cm areas within individual germination boxes. SeedRanger also supports programmable control over multiple imaging parameters‐including camera settings, capture frequency, temporal intervals, spatial coordinates, and sample throughput‐enabling fully experimental workflows.

In this study, the SeedRanger system acquired a total of 85,796 PNG format images documenting the germination processes of 356 cotton (*Gossypium spp*.) accessions, comprising 335 cultivated varieties, 5 wild cotton and 16 landraces from globally representative cotton germplasm resources (Table S1), with each genotype represented by 104 seeds per germination chamber. Each original image was recorded in 2K resolution (6000 ×4000 pixels) with an average size of approximately 11 MB, culminating in a total raw image file of 934.33 GB.

Raw images were resized to 25% of their original resolutions to improve processing efficiency. The region of interest (ROI) was extracted based on the b channel of the CIE L*a*b* color space (i.e., Lab) using the Otsu thresholding [22], followed by the erosion of mask boundaries to minimize background pixels (Figure S1a, left). Then, seed masks within the ROI were retained, with noises (e.g., due to reflections caused by water) removed based on ROI area and circularity [14] (Figure S1a, right). The first image of each image series was removed as too much water soaked by the germination paper could lead to noise. Seeds in the second image were indexed according to their centroid coordinates of the seed‐level masks, so that seed‐level tracking by overlapping all the images at consecutive timepoints in a given series could be performed (Figure S1b). Seed‐level morphological features, including seed area, seed length, seed width, seed perimeter, were quantified using the previously reported method [14] (Figure S1c), during the first 24 h (i.e. the imbibition stage). Radicles were segmented based on the L channel values of Lab, based on which 2D skeletons were obtained for length measurement over time and thus elongation rate calculation (Figure S1d, left). Green regions of cotyledon were quantified using the Excess Green index [23], localized by the intersection of seed coat regions. This was then followed by the quantification of green area and mean intensity values of the green channel pixels in the RGB color space (Figure S1d, right). During the seed‐level tracking process, the tracking algorithm was terminated when the following conditions occurred: (i) seeds touched the ROI boundary, (ii) seeds overlapped with other seeds, (iii) seeds failed to overlap with the same seed at consecutive timepoints, or (iv) seeds intersected with other seeds whose tracking had already been terminated (Figure S2). This process resulted in a total of 332 seeds being unrecognized (0.89% failure rate; Figure S3). Validation through linear regression analysis manually collected measurements against automated results demonstrated strong predictive accuracy, with coefficients of determination (*R^2^*) of 0.9285 (*n* = 103) for radicle length and 0.7351 (*n* = 103) for seed length, respectively (Figure S4). These results confirm the robustness of our computational approach for quantitative trait analysis in cotton germination study.

Overall, SeedRanger demonstrated exceptional performance in facilitating high‐throughput, precise monitoring of seed vigor throughout various developmental phases. The platform successfully acquired both static, standardized seed vigor characteristics and dynamic phenotypic expressions with superior temporal resolution. It achieved accurate individual seed tracking while extracting comprehensive digital traits that effectively represent real‐time germination dynamics.

### 2.2 High‐Resolution Phenotyping Reveals Dynamic Trajectories of Seed Vigor

**FIGURE 2:**
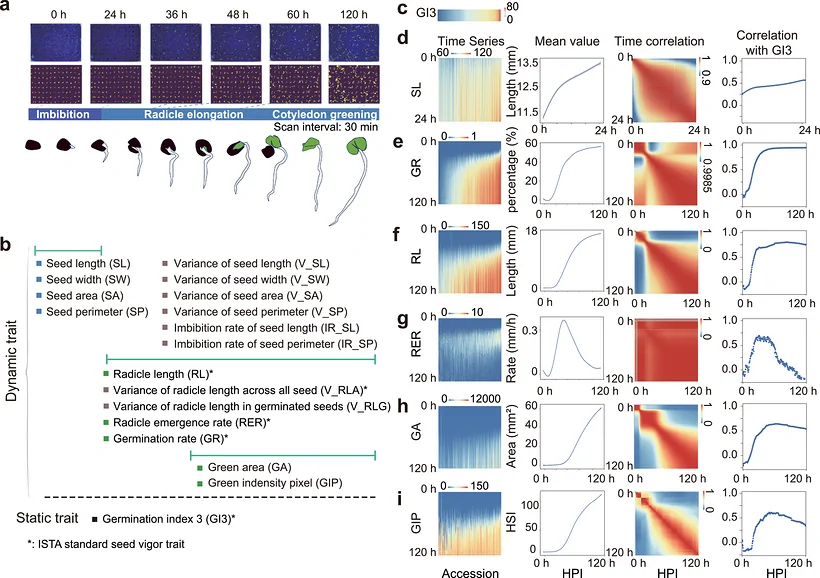
Dynamic image‐based traits (i‐Traits) captured during seed germination and their reliability assessment. a, Time‐lapse imaging were captured using the SeedRanger platform at intervals from 0 to 120 h after imbibition until the greening of cotyledons. b, Dynamic i‐Traits are presented based on the corresponding developmental stage of each seed. During the imbibition phase, four primary i‐Traits were recorded: seed length (SL), seed width (SW), seed area (SA), and seed perimeter (SP), along with four variance i‐Traits and two relative i‐Traits representing the imbibition rate of seed length (IR_SL) and seed perimeter (IR_SP). During the germination phase (24‐120 h post‐imbibition), five i‐Traits were recorded. At the onset of early chloroplast development, two i‐Traits were recorded: green area (GA) and green intensity pixels (GIP), indicating the emergence of green pigmentation in the seedlings. The germination index at 3 days (GI3) was collected as a traditional trait for assessing seed vigor. i‐Trait values at each time point represent the mean of 104 seeds per accession. c, Heatmap showing the seed vigor of different accessions, sorted by GI3 values. d‐i, Temporal dynamics and reliability analysis for six representative i‐Traits: seed length (SL; d), germination rate (GR; e), radicle length (RL; f), radicle emergence rate (RER; g), green area (GA; h), and green intensity pixels (GIP; i). For each trait (columns from left to right): heatmap of relative values across 356 accessions (sorted by GI3); mean temporal trajectory; temporal autocorrelation heatmap across all time points; and correlation dynamics with GI3 over time. All i‐Trait values represent the mean of 104 seeds per accession. *Denotes traits corresponding to the International Seed Testing Association (ISTA) standard for seed vigor.

Over a 120‐h period following imbibition, cotton seeds underwent three distinct developmental phases: imbibition (0‐24 h), radicle elongation, and cotyledon greening (Figure 2a). During the imbibition phase, key morphological parameters, including seed length (SL), seed width (SW), seed perimeter (SP), and seed area (SA), were quantified at both the dry seed stage (0 h) and at 24 h after imbibition to distinguish maternal seed size effects from post‐imbibition growth. Within‐accession variance for these traits (V_SL, V_SW, V_SP, V_SA) was calculated as the variance across individual seeds within each accession at each time point, using raw measured values without correction for accession means. Furthermore, imbibition rates were determined for seed length (IR_SL) and perimeter (IR_SP) (Figure 2b; Figure S1). Notably, although radicle elongation and cotyledon greening became visibly apparent around 24 h and 48 h post‐imbibition respectively, imaging commenced at 0 h to capture incipient physiological signals without temporal discontinuity. During the radicle elongation phase, radicle length (RL) was measured to derive the radicle emergence rate (RER) and two variance metrics: V_RLA represents the variance of radicle length calculated across all seeds (including non‐germinated seeds, assigned RL = 0), while V_RLG represents the variance calculated among germinated seeds only (RL > 0). Notably, both RER and V_RLA correspond conceptually to the germination vigor defined by ISTA protocols; specifically, RER quantifies the speed of germination emergence, whereas V_RLA captures the uniformity of germination across the entire seed population. Germination rate (GR) was also recorded at each time point (Figure 2b). In the cotyledon greening phase, the development of photosynthetic tissue was assessed by measuring the green area (GA) and green intensity pixels (GIP) of the cotyledons (Figure 2b; Figure S1). This comprehensive profiling yielded a set of 17 dynamic image‐based traits (i‐Traits) monitored continuously over 120 h. Additionally, Germination Index 3 (GI3), a conventional static seed vigor index, was included as a traditional reference benchmark (Figure 2b; Table S2). Key parameters such as radicle length, germination rate, radicle emergence rate, Variance of radicle length across all seed (V_RLA) and germination index were consistent with established ISTA seed vigor assessment protocols [1, 2, 7, 13, 24, 25]. Morphological traits, SL, SW, SP, and SA, increased consistently during the imbibition phase (Figure 2d, second column; Figure S6, second column). Similarly, GR, RL, GA, and GIP increased over time following appropriate germination periods (Figure 2e,f,h,i, second column). The RER exhibited a unimodal pattern, peaking between 36 and 48 h after imbibition (Figure 2g, second column). All i‐Traits demonstrated stronger temporal autocorrelation between adjacent time points compared to randomly sampled time points (Figure 2d–i, third column; Figure S6, third column), confirming the continuity of the phenotypic trajectories.

For comparative analysis and evaluation of the i‐Traits obtained, the traditional seed vigor trait GI3 was calculated. Overall, GI3 showed substantial phenotypic variation across the tested cotton population, with a mean of 29.55% (±16.89 SD) and an interquartile range (IQR) of 24.83% (Figure S5). Accessions were ordered by increasing GI3 values for visualization of i‐Traits (Figure 2c; Figure 2d–i, first column; Figure S6, first column). Accessions with higher GI3 values generally exhibited larger seed dimensions (Figure 2d, first column; Figure S6a‐c, first column), higher germination rates (Figure 2e, first column), longer radicles at comparable time points (Figure 2f, first column), an earlier and more concentrated peak in RER (Figure 2g, first column), and earlier and more intense cotyledon greening (Figure 2h,i, first column). Correlation analysis between GI3 and the i‐Traits revealed a strong positive association for germination rate (GR, *R* = 0.78) and radicle length (RL, *R* = 0.63) (Figure 2e,f, fourth column). The RER showed a moderate correlation (Figure 2g, fourth column), while variance‐based traits (V_RLG, V_RLA) exhibited intermediate correlation strengths (Figure S6j,k, fourth column). Seed size traits during imbibition (SL, SW, SA, SP) were moderately positively correlated with GI3 (Figure 2d, fourth column; Figure S6a‐c, fourth column), although their variance components and imbibition rates showed weak or non‐significant relationships (Figure S6d‐i, fourth column). Cotyledon greening associated i‐Traits (GA and GIP) displayed marginal positive correlations with germination performance (Figure 2h,i, fourth column). Collectively, these assessments indicate that the i‐Traits obtained from SeedRanger were overall associated with seed vigor index GI3, supporting their utility for dissecting the complex trait.

### 2.3 I‐Traits for Seed Vigor are Under High Impact of Genetic Control

**FIGURE 3:**
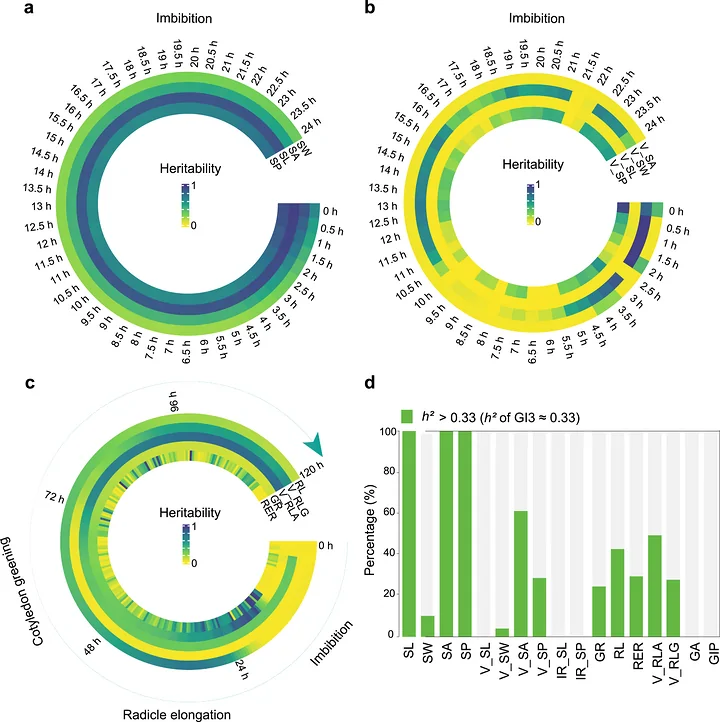
The complexity of seed vigor is represented by i‐Traits over a time series with dynamics in heritability. a‐b, Circle heatmaps showing heritability (*h^2^*) dynamics during the imbibition phase, for seed morphological traits (a: SL, SW, SA, SP) and their variance metrics (b: V_SL, V_SW, V_SA, V_SP). Color intensity (blue to yellow) and ring width indicate heritability values (0‐1). c, Circular heatmap showing heritability trajectories of germination and seedling traits (RL, RER, V_RLA, V_RLG, GA, GR) across the full 120‐h time course, with approximate developmental transitions indicated (imbibition → radicle elongation → cotyledon greening). The arrow indicates the chronological progression from 0 h to 120 h. d: Percentage of time points with heritability exceeding that of the GI (*h^2^* of GI ≈ 33%) for each i‐Trait. Green bars indicate i‐Traits with superior genetic power compared to GI3.

Narrow‐sense heritability (*h^2^*) quantifies the proportion of phenotypic variance attributable to additive genetic effects within a specific population. To investigate the genetic architecture of seed vigor‐related i‐Traits, the *h^2^* for each i‐Trait at every imaging time point during seed germination were calculated, along with the *h^2^* for GI3, which was approximately 33% (Figure 3a–d).

During the imbibition, morphological traits (SL, SA, SP) maintained higher *h^2^* than GI3 throughout the 0–24 h window, though their variance components fluctuated over time (Figure 3a,b). In the radicle elongation phase, dynamic traits including GR, RL, RER, V_RLA, and V_RLG exceeded GI3 *h^2^* for 20% to 50% of the monitoring period. Specific temporal windows were 15.5–44.5 h for GR, 23.5‐74.5 h for RL, 27–78 h for RER, 62–120 h for V_RLA, and 87–120 h post‐imbibition for V_RLG (Figure 3c; Figure S7).

Temporal dynamics of *h^2^* revealed distinct patterns: RL heritability showed a pronounced increase coinciding with radicle emergence (20‐24 h post‐imbibition), while GR displayed a similar but earlier onset of heritability elevation (Figure 3c; Figure S7). Notably, i‐Traits including RL and GR attained maximum *h^2^* values of 73.41% and 94.70%, respectively, during the 24–72 h germination window (Figure 3c; Figure S7), indicating strong genetic regulation of these i‐Traits at critical developmental stages. Throughout most of the experiment, seed vigor‐related i‐Traits showed significantly higher *h^2^* than the GI3 (Figure 3d). Combined with their strong phenotypic correlations to GI3 (Figure 2d–i, fourth column; Figure S6), these results confirm that dynamic i‐Traits are under robust genetic control and offer superior power for genetic dissection.

### 2.4 High‐Resolution Temporal Phenotyping Reveals Dynamic Genetic Architecture of Seed Vigor

**FIGURE 4:**
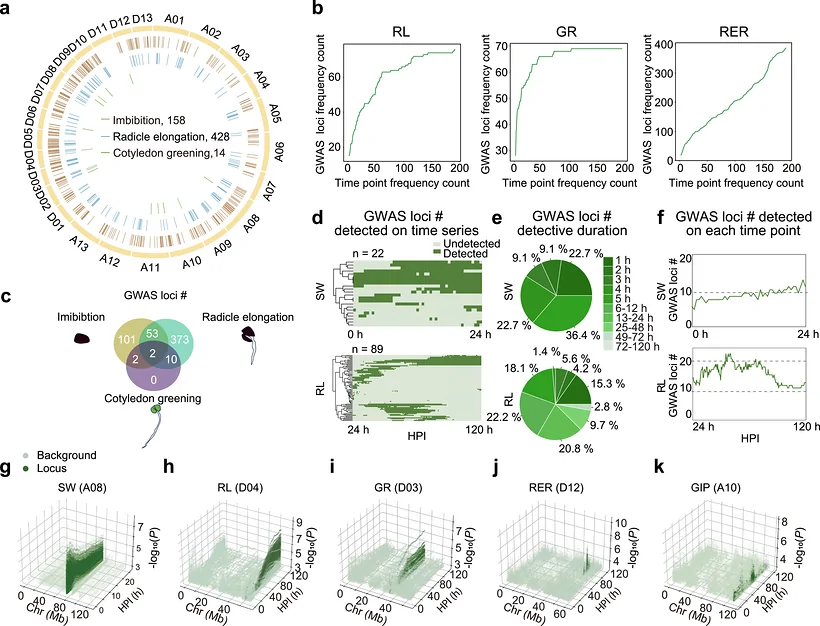
Genome‐wide association study of high‐throughput dynamic i‐Traits for seed vigor. a, Circular Manhattan plot showing the genome‐wide distribution of significantly GWAS loci for all i‐Traits across chromosomes. b, Cumulative frequency distribution of GWAS loci. The x‐axis represents the number of time points sampled; the y‐axis shows the cumulative number of GWAS loci detected. c, Venn diagram illustrating the overlap of GWAS loci identified at each developmental stage. d, Detection matrix of GWAS loci for seed width (SW, top, n = 22) and radicle length (RL, bottom, n = 89) across the time course. Dark green indicates a significant locus was detected at a given time point, while light green indicates none was detected. e, Pie charts showing the distribution of GWAS loci for SW (1–24 h) and RL (1–120 h) by detection duration. f, Line plot showing the number of GWAS loci detected at each time point throughout the monitoring period for SW and RL. g–k, 3D Manhattan plots showing dynamic GWAS signals over time for five i‐Traits: (g) seed width (SW, A08), (h) radicle length (RL, D04), (i) germination rate (GR, D03), (j) radicle emergence rate (RER, D12), and (k) green intensity pixels (GIP, A10). The *x*‐axis represents chromosomal position (Mb), the *y*‐axis represents time, and the *z*‐axis shows the ‐log_10_(*P*) for SNP‐trait associations, and Highlighted data points indicate the positions of significant GWAS loci for each i‐Trait.

An integrated genomic and temporal phenotyping analysis was performed for 356 cotton accessions, combining genome resequencing data with high‐resolution germination imaging (Table S1). Population genomic analysis identified 5,611,383 high‐quality SNPs, revealing four distinct geographical groups with significant differences in seed size and germination traits. Genome‐wide association studies (GWAS) at multiple significance thresholds identified 541 non‐redundant genetic loci significantly associated with i‐Traits through LD‐based clustering at the 3.98×10^−^^6^ threshold (Figure 4a; Table S3).

Notably, temporal resolution profoundly influenced detection sensitivity, as shorter sampling intervals (1–48 h) significantly increased locus identification rates, demonstrating the critical importance of high‐frequency phenotyping for capturing dynamic genetic effects (Figure 4b; Figure S9). Functional characterization revealed distinct locus categories, including 158 for seed imbibition, 438 for radicle elongation, and 14 for cotyledon greening, with 55 showing pleiotropic effects on both seed imbibition and radicle elongation (Figure 4c; Table S3).

High‐resolution mapping at 30‐min intervals allowed precise quantification of locus activity durations, revealing extensive temporal overlap among functional loci across development (Figure 4d; Figure S10b‐p). Locus activity was highly stage‐specific, as 63.6% of SW‐associated loci remained activate for under 4 h whereas 66.8% of RL‐associated loci persisted for less than 12 h (Figure 4e). Developmental stage profoundly influenced genetic architecture, with imbibition stages involving 8–10 loci per i‐Trait per timepoint, increasing to 10–20 loci during later germination phases (Figure 4f).

3D Manhattan plots visualized these temporal dynamics of locus activation. RL‐associated loci activated around 40 h post‐imbibition with gradually increasing significance, while RER‐associated loci exhibited narrow, well‐defined active windows at 30–40 h (Figures 4g‐k; Figure S11). These dynamic QTLs (dQTLs) provide evidence for temporally coordinated genetic regulation of germination, underscoring the complex temporal architecture underlying seed vigor.

### 2.5 Pleiotropic Loci and Candidate Genes Define a Genetic Network Controlling Seed Vigor

**FIGURE 5:**
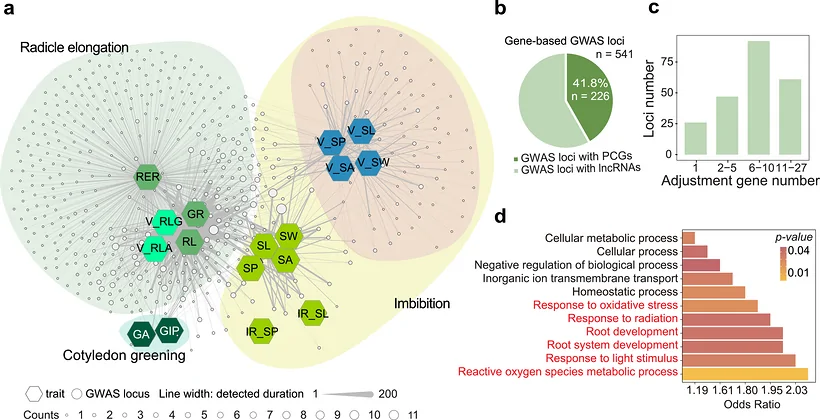
Integrated high‐throughput phenomics reveals a genetic regulatory network for seed vigor. a, Network visualization of seed vigor dynamic QTLs (dQTLs) identified through i‐Trait GWAS. Hexagons represent i‐Traits colored by developmental stage with line width indicating detection duration (1–200 time points). Small circles represent individual GWAS loci, sized by allele counts (1–11). Lines connect loci to their associated traits. b, Genomic distribution of gene‐based GWAS dynamic QTL loci (n = 541), showing the proportion associated with protein‐coding (PCGs; 41.8%, n = 226) versus long non‐coding regions (lncRNAs, 58.2%, n = 315). c, Distribution of adjustment gene numbers (regulatory genes within GWAS loci). d, Gene Ontology (GO) enrichment analysis of protein‐coding genes (PCGs) associated with seed vigor.

Genetic analyses revealed extensive pleiotropy on seed vigor regulation, with individual loci frequently influencing multiple phenotypic traits. Specifically, 55 loci demonstrated consistent effects across both imbibition and radicle elongation stages. Notably, loci associated with cotyledon greening substantially overlapped with those regulating imbibition and post‐germination processes, suggesting that these continuous developmental stages share conserved genetic pathways. This pleiotropy was further evidenced by the co‐localization of loci controlling seed size and imbibition efficiency with those governing radicle elongation and germination rate (Figure 5a).

Distinct genetic architectures were uncovered through developmental‐stage‐specific clustering. Radicle elongation loci formed overall isolated clusters from imbibition loci, and the radicle elongation stage exhibited greater genetic complexity, with increased locus numbers and prolonged activity durations corresponding to its dynamic physiological state (Figure 5a). These patterns indicate that seed vigor is regulated through an interconnected genetic network.

To elucidate molecular mechanisms underlying this network, the potential functional genes in the seed vigor genetic network were characterized by gene‐based association methods [26]. A total of 1,555 candidate genes were identified from 226 of the 541 GWAS loci (Figure 5b; Tables S3 and S4). Most loci (41.8% of total GWAS loci) contained fewer than 10 associated genes, while the remaining loci likely reside in non‐coding regions, as evidenced by the absence of protein‐coding genes within their LD blocks (Figure 5c).

Gene Ontology (GO) enrichment analysis revealed significant overrepresentation of biological processes critical for seed germination, including oxidative stress response, root system development, and reactive oxygen species metabolism (Figure 5d; Table S5). Notably, *HSP24.7* was identified among these candidates, representing, a known germination activator previously documented in cotton (Figure S12) [27].

### 2.6 A Dynamic QTL Reveals FLA2 as a Pleiotropic Regulator of Seed Germination

**FIGURE 6:**
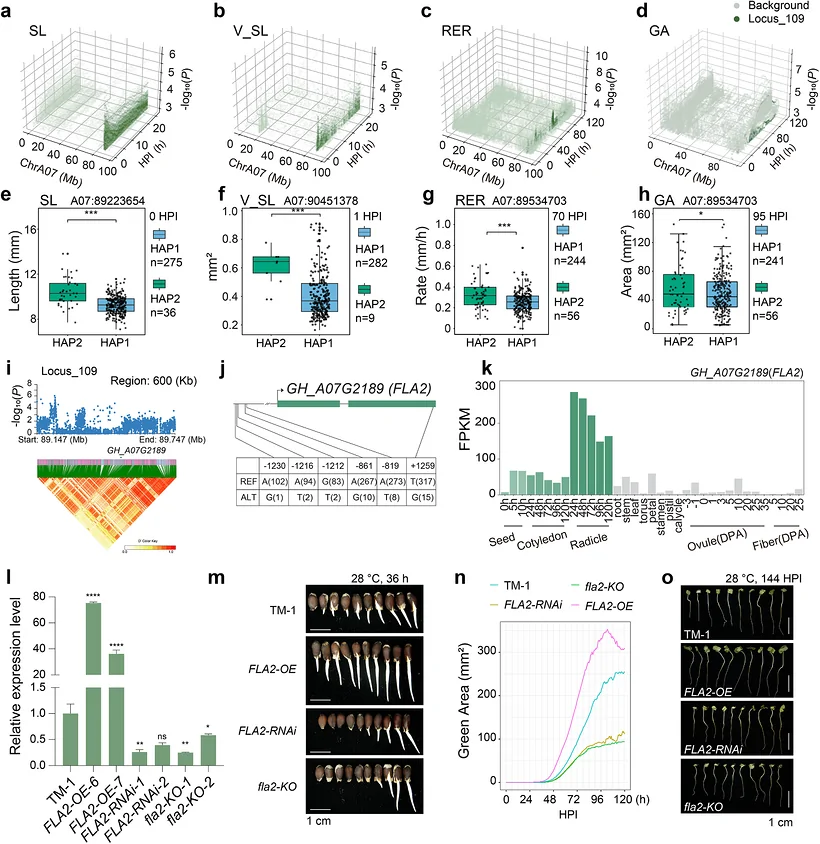
Gene‐based GWAS identifies FLA2 as a candidate gene regulating seed vigor at Locus_109. a‐d, 3D Manhattan plots of Locus_109 across the imbibition and radicle elongation time series for the i‐Traits seed length (SL; a), variance of seed length (V_SL; b), radicle emergence rate (RER; c), and green area (GA; d). e‐h, Haplotype effects of Locus_109 on SL (e), V_SL (f), RER (g) and green area (GA, h). Data are presented as mean ± s.d.; *p < 0.05, **p < 0.01, ***p < 0.001, by two‐sided Student's *t‐*test. i, Manhattan plot (top) and linkage disequilibrium (LD) heatmap (bottom) for seed length at 0 h (SL, start of imbibition) with the Locus_109 (A07: 89,147,223–90,632,366), highlighting the candidate gene GH_A07G2189/FLA2 (A07: 89,504,596–89,505,858). j, Schematic diagram of the *FLA2* gene structure and the SNPs identified across the promoter and gene region. k, Tissue‐specific expression profile of *FLA2* showing FPKM values across various cotton tissues. l, Relative expression of *FLA2* in transgenic cotton lines: overexpression (*FLA2‐OE*), RNA interference (*FLA2‐RNAi*), and knockout (*fla2‐KO*). Wild‐type (TM‐1) serves as the control. Data are mean ± s.d.; *p < 0.05, **p < 0.01, ****p < 0.0001 by two‐sided Student's *t*‐test; n ≥ 3 biologically independent samples. m, Radicle length phenotypes of TM‐1, *FLA2‐OE*, *FLA2‐RNAi*, and *fla2‐KO* lines at 36 h post‐imbibition (36 HPI, 28°C). Scale bar, 1 cm. n, Temporal dynamics of the i‐Trait green area (GA) in TM‐1, *FLA2‐OE*, *FLA2‐RNAi*, and *fla2‐KO* throughout the germination time course (0‐120 h), as quantified by SeedRanger. n = 104. o, Representative images of germinating seeds from TM‐1, *FLA2‐OE*, *FLA2‐RNAi*, and *fla2‐KO* after 144 h post‐imbibition (144 HPI, 28°C). Scale bar, 1 cm.

Our analysis of the dynamic genetic architecture of seed vigor identified Locus_109 (A07: 89,147,223–90,632,366) as a key pleiotropic locus operating across imbibition, radicle‐elongation, and cotyledon greening stages. This locus showed significant associations with seed length dynamics during imbibition (encompassing seed size effects at 0 h and post‐imbibition growth at 24 h), as well as variance of seed length (V_SL), radicle emergence rate (RER), green area (GA) (Figure 6a–d; Figure S13). Figure 6i displays the Manhattan plot for seed length at 0 h (SL, start of imbibition) within this shared time interval. Slightly distinct association patterns at radicle‐elongation of RER and cotyledon greening (GA) were also observed (Figure 6c,d). Haplotype analysis of Locus_109, defined by trait‐associated SNPs, revealed significant phenotypic differences between accessions with the favorable haplotype (HAP1) and those with the alternative haplotype (HAP2) (Figure 6e–h). Furthermore, Locus_109 is overlapped with an eGene *GH_A07G2189/FLA2* (A07: 89,504,596–89,505,858) (Figure 6i), previously reported eQTLs for 1‐DPA ovule expression, boll weight, and seed index [28]. Figure 6j illustrates the SNPs identified across the promoter and gene region of *FLA2*. Within this multi‐trait QTL interval, we prioritized *GH_A07G2189/FLA2* as the candidate gene based on its physical position overlapping the consensus QTL region, preferential expression in radicles (Figure 6k), and colocalization with established yield‐related eQTLs.

To functionally validate *FLA2* in seed vigor, we generated transgenic materials including overexpression (*FLA2‐OE*), RNA interference (*FLA2‐RNAi*), and CRISPR‐Cas9 knockout (*fla2‐KO*) lines (Figure S14; Figure 6l). At comparable developmental stages of 36 h post‐imbibition (HPI), *FLA2‐OE* lines exhibited accelerated radicle emergence, whereas suppression or knockout of *FLA2* significantly delayed radicle development (Figure 6m). This established that genetic perturbation of *FLA2* directly alters root system initiation dynamics. We next examined whether this altered root development was coupled with changes in photosynthetic tissue establishment. Time‐course analysis of seedling development revealed that enhanced radicle elongation in *FLA2‐OE* lines was synchronized with promoted cotyledon expansion, as quantified by green area (GA), while suppressed *FLA2* expression caused coordinated defects in both root and shoot development (Figures 6n,o; Figure S15 and Video S2).

Transcriptomic analysis indicated that *FLA2* regulates seedling vigor through upregulation of *IAA18* (Figure S16), a key component of the auxin signaling pathway with conserved functions in seedling and root development [29, 30, 31]. These findings establish *FLA2* as a key regulator of seed germination and provide a theoretical foundation for its application in developing high‐vigor cotton varieties, though its performance under sub‐optimal abiotic stresses (e.g., chilling, salt or osmotic stress) requires future investigation.

### 2.7 Domestication History Reveals Selection Signatures on Seed Vigor Traits in Upland Cotton

**FIGURE 7:**
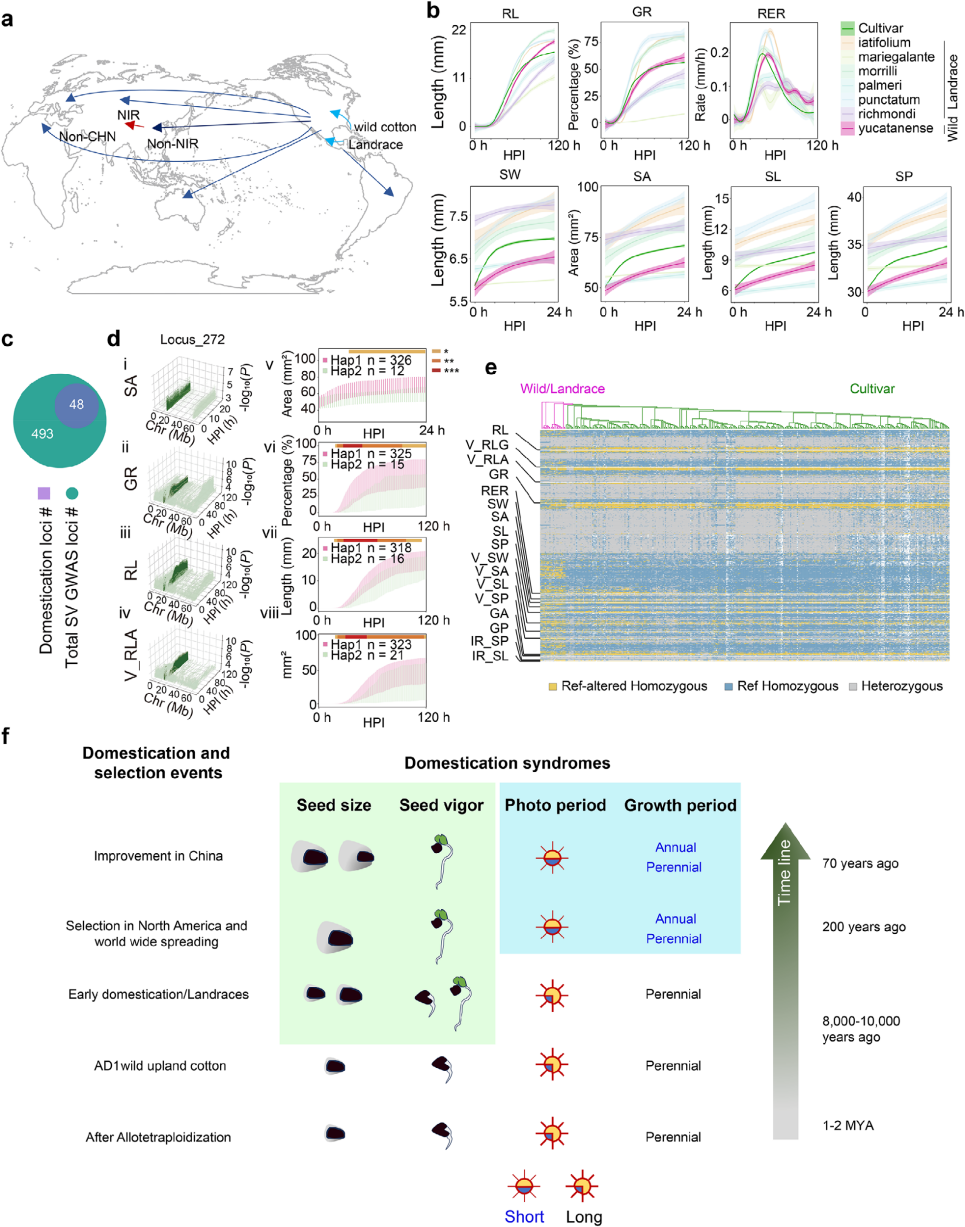
Seed vigor QTLs exhibit signals of selection during cotton domestication. a, Global dissemination routes of upland cotton during domestication. Base map source: Standard Map Service System, Ministry of Natural Resources of China (https://bzdt.ch.mnr.gov.cn), approval number GS(2016)1611. The original fill and boundary colors were inverted for visualization purposes; no boundaries were modified. b, Temporal dynamics of seed vigor i‐Traits (RL, GR, RER, SW, SA, SL, SP) across wild, landrace, and cultivated cotton groups, showing progressive enhancement of vigor‐related phenotypes during domestication. c, Venn diagram illustrating the between seed vigor GWAS loci and domestication selection regions. A total of 48 loci (8.9%) colocalize with domestication sweeps (*F_ST_* > 0.57 and *θ*_π_ ratio < 0.85) out of 541 significant GWAS loci. d, multi‐trait association and haplotype analysis at Locus_272. (i‐iv) Three‐dimensional Manhattan plots illustrating the dynamic association of Locus_272 with SA, GR, RL, and V_RLA across the time series. (v‐viii) Temporal trajectories of the corresponding i‐Traits comparing HAP1 and HAP2. *p < 0.05, **p < 0.01, ***p < 0.001, by two‐sided Student's *t‐*test. e, Haplotype distribution of seed vigor GWAS loci across the CUCP2 panel. Each row represents a locus, and each column represents an accession. f, Schematic timeline of selection for seed vigor traits compared with key domestication syndromes, including seed size, photoperiod sensitivity, and growth period.

*Gossypium hirsutum* L. originated in the Caribbean region approximately 8,000–10,000 years ago [32]. Over the past two centuries, cultivation expanded globally, demonstrating adaptation from short‐day equatorial to long‐day temperate environments (Figure 7a). Although early domestication focused primarily on fiber yield, modern cultivars exhibit improved photoperiod sensitivity, plant architecture, and growth habit. However, the extent to which seed vigor traits were selected during this process remains poorly understood.

The population analyzed in this study captures broad genetic diversity, including: (1) 5 wild *yucatanense* accessions, (2) 16 distinct landraces, (3) 86 accessions from the Northwest Inner Region of China (NIR CHN), (4) 145 accessions from non‐NIR CHN regions in China, and (5) 104 accessions from outside China (Figure S8a–c; Table S1). The non‐China subgroup comprises germplasm from the United States, former Soviet Union territories, and other major cotton‐producing regions. Genomic analysis incorporated resequencing data from 349 previously studied accessions, supplemented with seven newly sequenced accessions (Table S1).

To evaluate the impact of domestication on seed vigor, we compared wild (*yucatanense*), landrace, and modern cultivated varieties. i‐Trait analysis revealed significant differences (*p* < 0.001, Student's *t*‐test) in seed dimensions, length, width, perimeter, and area, during imbibition (Figure S8d). *Yucatanense* exhibited significantly lower seed vigor relative to domesticated cultivars (*p* < 0.01), with the phenotypic disparity increasing during later imbibition stages (Figure 7b). Landraces displayed intermediate phenotypic variation in seed morphology and vigor, with significantly higher coefficients of variation than modern cultivars (Figure 7b), consistent with artificial selection for increased seed size and improved viability during domestication.

Genomic signatures have reinforced these phenotypic patterns. Notably, 8.9% (n = 48) of seed vigor associated GWAS loci overlapped with domestication selection regions defined by stringent statistical thresholds (Figure 7c; Table S6). *F_ST_* and *θ*_π_ have proven highly effective for detecting selective sweep regions. A sliding window approach was applied using 100‐kb windows with 10‐kb steps. domestication candidates were identified as genomic windows with both high population differentiation (*F_ST_* > 0.57, top 5% genome‐wide) and reduced nucleotide diversity in cultivated accessions relative to their wild progenitors (*θ*_π_ ratio < 0.85, bottom 5%; *θ*_π_ ratios = (*θ*_π, cultivated_/*θ*_π, (wild+Landrace)_) (Figure S17). These 48 loci were concentrated within domestication sweeps, suggesting that seed vigor traits have been subject to selection during cotton domestication. Detailed *F_ST_* and *θ*_π_ ratio values for all genomic intervals, along with their percentile rankings (top 5% *F_ST_*, bottom 5% *θ*_π_ ratio) and overlap status with GWAS loci, are provided in Table S6. For example, Locus_272 illustrates how selection acted sequentially across developmental stages, from seed imbibition to cotyledon greening, to enhance seed vigor (Figure 7d; Figure S18a). Furthermore, seed vigor QTL mapping clearly differentiated wild, landrace, and modern varieties (Figure 7e), demonstrating that seed vigor has been a target of selection during cotton improvement and highlighting how domestication shaped this agronomically essential trait complex.

## 3 Discussion

### 3.1 Seed Vigor as a Key Breeding Target for Mechanized Agriculture and Evidence of Its Domestication Selection

From an agronomic perspective, enhancing seed vigor has become a central breeding objective, directly supporting the modernization and mechanization of crop production systems. To optimize yield and minimize costs, mechanized farming requires seeds to be strictly uniform in size, shape and quality, which is a clear calling for high seed vigor. Crop domestication has led to significant improvements in yield performance, seed vigor, and environmental adaptability to meet global agricultural demands. In response to growing demands for mechanized production, there is an urgent need for breeding programs to simultaneously improve seed vigor and phenotypic uniformity. The development of high‐vigor seed stocks is fundamental for achieving synchronized crop growth stages, and elucidating the underlying genetic mechanisms represents a core scientific priority for advancing mechanized agriculture and ensuring global food security.

This study provides clear evidence that seed vigor has been an independent target of cotton domestication. Beyond its well‐documented roles in fiber yield and adaptation, our analysis demonstrates that seed vigor was under direct selection during cotton improvement, which may be earlier than the domestication of photo period in landrace (Figure 7b–f). Significant enrichment of GWAS loci within domestication regions, coupled with the progressive differentiation in vigor among wild, landrace, and modern varieties, indicates that early and uniform germination traits were consistently favored. The temporal pattern of selection signals, spanning from imbibition to seedling establishment (Figure 7f), suggests that human selection optimized the entire developmental sequence rather than individual components.

Our results indicate that during the initial phases of upland cotton domestication, selective pressures primarily targeted yield enhancement and seed vigor to ensure production stability. As cultivation expanded to higher latitudes, key domestication traits such as photoperiod insensitivity and compact plant architecture were progressively fixed. Notably, selection on seed vigor manifested earlier in the domestication timeline than selection for photoperiod response and plant architecture modifications (Figure 7f), further establishing seed vigor as a key component of the domestication syndrome.

### 3.2 High‐temporal‐resolution Phenotyping Reveals the Dynamic Genetic Architecture of Seed Vigor

Plant phenomics has emerged as a powerful framework for investigating crop populations and elucidating the genetic architecture of seed vigor through identifying major quantitative trait loci (QTLs) and functional genes. This high‐throughput phenotyping approach has demonstrated broad applicability across diverse crop species [16, 33, 34, 35, 36, 37, 38, 39, 40, 41, 42, 43, 44, 45, 46, 47, 48, 49, 50, 51, 52, 53], underscoring the critical importance of temporal phenotypic dynamics in plant breeding [33, 35, 37, 41, 54, 55].

The temporal dimension substantially augments the complexity of biological process research by amplifying heterogeneous variability across tissue levels. Our experimental design incorporated population‐level detection parameters and high‐resolution temporal sampling (30‐min intervals) within a condensed timeframe, enabling synchronous monitoring of germination dynamics across multiple cotton accessions while minimizing measurement error and maximizing genetic resolution.

Our findings also indicate that seed vigor is not a static endpoint but a dynamically orchestrated trait governed by sequential genetic programs [14, 56]. The extracted phenotypic parameters showed strong temporal autocorrelation, and the continuity observed in root elongation trajectories, germination kinetics, and seed size heritability collectively validated the comprehensiveness of seed‐germination phenotyping. Fluctuations in heritability during germination—with distinct peaks corresponding to key developmental transitions—demonstrated that genetic control operates in stage‐specific modules. This temporal partitioning of phenotypic variance allowed us to identify dQTLs with precise activation windows, revealing that brief, transient genetic effects collectively drive germination outcomes. Furthermore, our i‐Traits system exhibited significantly enhanced heritability estimates throughout germination, substantially exceeding the genetic power of static measurements such as the germination index. This aligns with the broader paradigm shift toward AI‐driven seed quality analysis, where automated imaging and predictive algorithms are progressively replacing labor‐intensive traditional assessments [57]. Notably, dynamic i‐Traits attained maximum *h^2^* values of 73.41% (RL) and 94.70% (GR) during the 24–72 h window, compared to only ∼33% for GI3. This study provides the first report of temporal patterns in phenotypic heritability dynamics during development, confirming the reliability of i‐Traits for genetic dissection. Together, these results establish temporal phenomics as an essential paradigm for dissecting time‐dependent complex traits, shifting the focus from static snapshots to continuous biological processes.

### 3.3 The Pleiotropic Nature of Genetic Loci Regulating Seed Vigor can be Exploited to Simultaneously Improve Seed Yield and Adaptability

Furthermore, our findings reveal that seed vigor‐associated loci frequently exhibit pleiotropy, enabling simultaneous optimization of multiple aspects of germination and early growth. Loci controlling seed size, imbibition rate, and radicle elongation show genomic colocalization, indicating they were co‐selected as functional units. This pleiotropic architecture facilitates coordinated improvement of seedling robustness‐related traits while reducing unfavorable linkages. The concentration of selection signatures in these multi‐Trait hubs underscores their central role in aligning seed performance with agronomic requirements.

Furthermore, the high‐heritability i‐Traits identified here, particularly those associated with emergence uniformity and radicle elongation, provide robust targets for breeding, but their genotype × environment (G×E) interactions represent a critical next step for translation to field conditions.

One important aspect of seed vigor assessment is the tolerance of the germination process to various stresses. Although our technical pipeline provided relatively optimized and consistent conditions for population‐level germination, and therefore did not, in a strict sense, evaluate germination performance under adverse conditions, true phenotyping of seedling‑stage stress traits requires purpose‑built experimental designs and dedicated phenotyping and evaluation systems. Seed vigor encompasses multiple developmental stages, from imbibition and radicle emergence to chloroplast biogenesis, and our study identified numerous genetic loci with pleiotropic effects on development, dormancy and germination rate. Because these functions are tightly linked to hormone signaling and reactive oxygen species (ROS) pathways, such loci may also have potential to enhance seedling stress adaptation. Consequently, while the current study provides the foundational genetic architecture under optimal conditions, multi‐environment testing is essential to confirm whether *FLA2* and other dQTLs retain their effects across the stress gradients encountered in mechanized production systems.

### 3.4 Environmental Context and Limitations of the Current Study

While the current study establishes the genetic architecture of seed vigor under controlled greenhouse conditions (28°C), we acknowledge that ISTA defines seed vigor as performance across diverse environments, including sub‐optimal conditions such as chilling or soil crusting [58]. Our standardized phenotyping provides a baseline characterization of genetic potential under optimal germination conditions, distinct from stress tolerance assessment which requires dedicated experimental designs.

Regarding the stability of our findings under abiotic stress, *FLA2* warrants particular attention. Located within the pleiotropic Locus_109 that operates across imbibition, radicle‐elongation, and cotyledon greening stages, *FLA2* regulates seedling vigor through upregulation of *IAA18*, a key component of the auxin signaling pathway. Auxin signaling pathways, including regulators such as *IAA18*, are known to mediate adaptive responses to both salt and osmotic stress [59, 60], suggesting that *FLA2*‐mediated radicle emergence may retain functional relevance under field stress conditions. Nevertheless, we acknowledge that this auxin‐mediated mechanism is currently inferred from gene expression correlation and requires future direct experimental validation. However, whether the specific allelic effects observed here persist under sub‐optimal environments remains experimentally untested. Future research necessitates standardized stress phenotyping platforms to systematically verify the functional stability of these allelic effects under sub‐optimal field conditions.

The high‐heritability i‐Traits identified here provide robust targets for breeding, but their genotype × environment interactions require validation across field‐relevant stress gradients [61]. Multi‐environment testing is essential to confirm whether FLA2 and other dQTLs maintain their effects under the chilling and soil crusting conditions encountered in mechanized production systems.

## 4 Material and Methods

### 4.1 Plant Materials and Growth Condition

To capture the genetic diversity of cotton germplasm, we assembled a collection of 356 cultivated (*Gossypium hirsutum*), landrace and wild cotton accessions from major cotton‐producing regions, including China, the United States, the former Soviet Union, Brazil, and other key production areas (Table S1).

Standardized seed germination phenotyping under controlled conditions: Seed germination assays were carried out during the 2022 growing season using the SeedRanger platform at the facility of Hangzhou Liquan Technology Co., Ltd. (Hangzhou, China). Cotton seeds were placed in uniformly sized germination chambers with equal volumes of distilled water. All experiments were performed under controlled greenhouse conditions: under dark conditions with temperature maintained at a constant 28°C (±0.5°C) for 120 h, following established ISTA seed vigor testing protocols.

### 4.2 Seed Processing and Storage

All cotton accessions were cultivated at the experimental station in Sanya, Hainan Province, China, with planting initiated in November 2020. Self‐pollinated seeds were manually harvested in March 2021. Following ginning, seeds retaining short fibers were subjected to acid delinting using concentrated sulfuric acid (specific gravity 1.84) at room temperature with continuous mechanical stirring until complete lint removal (approximately 3–5 min). The acid was rapidly diluted with excess distilled water, and seeds were immediately subjected to density‐based separation: floating seeds (including cracked, immature, or damaged individuals) were discarded, while intact seeds settling at the bottom were retained. Selected seeds were transferred to nylon mesh bags and washed under running tap water with vigorous hand scrubbing to remove residual fibers and ensure complete acid removal, followed by a 10‐min final rinse. Seeds were air‐dried at ambient temperature (20‐30°C) to standard moisture content and subsequently stored in a controlled seed storage cabinet until used for germination assays.

### 4.3 High‐throughput Dynamic Monitoring of Seed Vigor Using the SeedRanger Platform System Configuration

The SeedRanger platform integrates three core components: an automated germination chamber, an optical imaging system, and a robotic positioning system (Figure 1). Each germination chamber consists of a 30 × 40 cm polypropylene container with an automated lid system to maintain consistent humidity. Chambers are mounted on a modular cultivation rack with 60 cm vertical spacing between tiers.

Image acquisition is performed using a Canon EOS 77D RGB camera powered via a regulated supply. The camera is mounted on a T‐configuration robotic manipulator (Video S1) equipped with precision linear tracks, providing a maximum payload of 25 kg and positional repeatability of ± 0.02 mm. A complete imaging cycle across 27 predefined positions is completed within 7 min.

### 4.4 Germination Protocol

For standardized germination assessment, all germination boxes were prepared by lining them with 3‐mm‐thick germination paper. Seeds of each cotton accession (104 seeds per genotype) were systematically arranged in a grid pattern with 1.5 × 2 cm spacing between adjacent seeds, and each box was assigned a unique 2D barcode for identification. The germination process was initiated by adding 1 L of distilled water to each box, with strict temporal consistency maintained across all boxes (≤7 min variation in watering times). Throughout the 120‐h experimental period, germination proceeded under tightly controlled dark conditions at a constant temperature of 28°C (±0.5°C). To minimize environmental disturbances, chamber lids were opened exclusively during the brief imaging sessions. The experimental design processed 27 accessions per run, with high‐frequency image acquisition performed at 30‐min intervals, yielding a total of 85,796 PNG images for subsequent analysis.

### 4.5 Computational Vision for i‐Trait Conversion

Raw images were down sampled to 25% resolution for processing efficiency. The region of interest was identified via Otsu thresholding in the CIE L*a*b* color space [22], with seed masks retained after area and circularity‐based noise removal [14]. Seed tracking was initiated from the second image of each series based on centroid coordinates, with termination criteria including ROI boundary contact, seed overlap, or tracking failure. Morphological traits (area, length, width, perimeter) were quantified during imbibition (0–25 h) using the previously reported method [14]. Radicles were segmented using L‐channel values, with skeletonization enabling length measurement and elongation rate calculation. Cotyledon greening was assessed via Excess Green index [23], with quantification of green area and intensity in RGB space.

### 4.6 Data Filtering and Processing

Morphological traits (seed length, width, area, and perimeter) and radicle length were tracked throughout germination, yielding approximately 0.86 million high‐quality measurements. To ensure data accuracy, we implemented a rigorous filtering pipeline addressing potential artifacts from water reflections and stains, which affected approximately 0.89% of root length measurements. The filtering protocol included: (1) exclusion of seeds that never germinated (all radicle length values = 0), and (2) removal of seeds showing non‐zero initial radicle length (indicative of water reflection artifacts). This processing yielded 17 validated i‐Traits for subsequent analysis (Table S2).

Radicle length was quantified via pixel counting and converted to physical measurements using calibrated standards. Germination rate was calculated as (number of germinated seeds / total seeds tested) × 100. Radicle elongation rate was determined as (Rad_[i]_ – Rad_[i‐1]_)/0.5, where i represents sequential 30‐min timepoints.

We computed standard germination indices using established formulas: Germination Index (GI) = Σ(Gt/Dt), where Gt represents the number of seeds germinated on day t, and Dt denotes the corresponding day number [60]. Based on germination kinetics analysis identifying 24–72 h as the peak germination window, we calculated the three‐day germination index (GI3) and germination potential as (number of seeds germinated during peak period / total seeds tested) × 100%.

### 4.7 Correlation Analysis of i‐Traits

Pairwise correlations among i‐Traits across all time points were computed using the cor() function in R (v4.2.0). Resulting correlation matrices were visualized as heatmaps using the pheatmap package (v1.0.12).

### 4.8 Detection of Genomic Variations

For GWAS analysis, 356 accessions were aligned to the upland cotton TM‐1 reference [63]. The selected population represents cotton cultivated globally and includes improvements made in China, particularly in the China Upland Cotton Population 2 (CUCP2) for seed vigor. The average sequencing depth was 8.85‐fold, with about 142 accessions having coverage exceeding 10‐fold (Table S1). Briefly, raw paired‐end reads were filtered by Trimmomatic (v 0.38) [62] with default parameters and subsequently mapped to the cotton reference [60] using the ‘mem’ algorithm of BWA (v 0.7.17‐r1188) [63]. Aligned reads were converted to BAM format using samtools (v 1.9) [64], and duplicate reads marked with the MarkDuplicates method of Picard (v 1.124) (http://broadinstitute.github.io/picard). Variants were called using samtools (v 1.9) with parameters ‘‐q 20 ‐Q 15 –ugf’ and bcftools (v 1.8) with parameters ‘vmO z’ [65]. Biallelic SNPs were selected for further analysis using vcftools (v 0.1.13) [66] with filter parameters of mapping depth > 3, mapping quality > 20, genotyping rate > 90% and minor allele frequency (MAF) > 0.05. Annotation of SNPs was performed using ANNOVAR (version 2015‐12‐14) [67] based on the reference genome annotation.

### 4.9 Population Structure Analysis

Phylip software (v 3.69) was used to generate the neighbour‐ joining tree, which was then visualized using Evolview (www. evolgenius.info/evolview) [68]. PCA was performed with GCTA (v 1.92.1) [69] using the SNP data. For genetic diversity analysis, vcftools (v 0.1.13) was used to calculate p and *F_ST_* with a window size of 100k bp and a step size of 100 kb [66].

### 4.10 Heritability Estimation

Narrow‐sense heritability was estimated using the GCTA‐GREML method [70]. Genetic relationship matrices were constructed for common haplotypes with GCTA (v1.92.1) using the ‘make‐grm’ option [69]. Phenotypic variance explained by SNP sets was assessed via the ‘mgrm’ approach. To ensure robustness, we performed 100 iterations of random SNP sampling, calculating heritability as V(G)/V(P) in each replicate.

### 4.11 Genome‐Wide Association Study

Genome‐wide association analysis was performed using 5,611,383 high‐quality SNPs with minor allele frequency > 0.05. We conducted association tests using Efficient Mixed‐Model Association eXpedited (EMMAX) software [71]. The genome‐wide significance threshold (*p* = 3.98 × 10^−^^5^) was determined by estimating the effective number of independent SNPs using GEC (v0.2) [72]. To define independent association signals, we performed linkage disequilibrium (LD)‐based clustering of significantly associated SNPs using PLINK (v1.90) [73] with parameters ‐r2 ‐ld‐window 99999, merging variants within LD blocks (*r^2^* > 0.1) into single loci. Candidate genes for each GWAS locus were identified through gene‐based association analysis implemented in GCTA [69].

### 4.12 Construction and Molecular Detection of Genetically Modified Materials

This study employed multiple genetic transformation strategies to generate transgenic cotton materials. For the *GhHSP24.7* gene, its full‐length coding sequence was cloned into the WMV062 vector to construct an overexpression vector with N‐terminal FLAG tag fusion driven by the 35S promoter. A 301‐bp cDNA fragment was selected to construct its knockdown vector. Meanwhile, two sgRNAs (ATATCCGGATTGAAATGCCGGGG and GGAGGATGTTAAGATCTCGGT‐GG) were designed using the CRISPR‐P platform [74] to establish a CRISPR/Cas9 genome editing system simultaneously targeting *GhHSP24.7_A* (*GH_A12G2559*) and *GhHSP24.7_D* (*GH_D12‐G2578*). For the *GhFLA2* gene, its full‐length coding sequence was cloned into the *Xba* I and *Sal* I sites of the pCambia2300‐3×Flag vector to construct an overexpression vector. The RNAi vector was designed with a hairpin loop structure: the RTM loop sequence was first cloned into the same restriction sites, followed by insertion of a 500‐bp coding region from the *GhFLA2* conserved domain and its reverse complementary sequence on either side of the RTM sequence to form a self‐complementary hairpin RNA structure. A double mutant was constructed using the CRISPR‐Cas9 system with two sgRNAs (GAACAGTAATGGTTTCGCGTCGG and GTAGCC‐AGAGGAACCAGGAGCGG) simultaneously targeting *GhFLA2_A07* (*GH_A07G2189*) and its homologous gene *GhFLA2_D07* (*GH_D07G2124*). All genetic transformations were performed in the upland cotton TM‐1 background and completed by WIMI Biotechnology Co., Ltd. using the shoot apical meristem cell‐mediated transformation system [75]. Mutations in the *GhHSP24.7* and *GhFLA2* gene‐edited lines were verified by constructing next‐generation sequencing libraries and analyzing the data through the Hi‐TOM platform [76].

### 4.13 RNA Isolation and RT‐qPCR

Total RNA was isolated from 36 h imbibition seeds of *GhHSP24.7* and *GhFLA2* transgenic lines and the wild‐type control TM‐1 using the SteadyPure Plant RNA Extraction Kit (AG21019; Accurate Biotechnology, Changsha, China), according to the manufacturer's instructions. First‐strand cDNA was synthesized from 2 µg of total RNA using the *Evo M‐MLV* RT Mix Kit with gDNA Clean for qPCR Ver.2 (AG11728; Accurate Biotechnology, Changsha, China) reverse transcription system. Quantitative RT‐PCR was performed with the SYBR Green Premix Pro Taq HS qPCR Tracking Kit (AG11733; Accurate Biotechnology, Changsha, China) to analyze target gene expression. Relative transcript levels were quantified using the comparative 2^^−ΔΔCt^ method. All experiments were performed with three independent biological replicates. RT‐qPCR primer sequences are provided in Table S7.

### 4.14 RNA‐seq Library Preparation and Bioinformatic Analysis

Total RNA was isolated from seeds of FLA2 transgenic lines collected at 36 h after imbibition. Sequencing libraries were generated and subjected to paired‐end sequencing (150 bp) on the DNBSEQ platform (BGI Genomics, Zhejiang Annoroad Bio‐Technology Co., Ltd., Jinhua, China). Raw reads were filtered to remove adapter sequences and low‐quality reads, and the resulting clean reads were aligned to the TM‐1 reference genome [61] using HISAT2 [77] Gene expression was calculated as FPKM values [78]. Differential expression analysis was conducted using DESeq2 [79] with thresholds of |log_2_FC| > 1 and adjusted *p*‐value < 0.05. Functional enrichment analyses, including GO and KEGG pathway analysis, were implemented using the R package clusterProfiler [80].

### 4.15 Identification of Domestication Selection Regions

To detect selective sweeps associated with cotton domestication, we scanned the genome in 100‐kb sliding windows with a step size of 10‐kb to calculate fixation index (*F_ST_*) and nucleotide diversity ratios (*θ*_π_ ratio = *θ*_π, Cultivar_/*θ*_π, (wild+Landrace)_) between wild/landrace and cultivated cotton accessions [81]. Genomic regions with significantly high differentiation (*F_ST_* > 0.57, corresponding to the top 5% right tail of the genome‐wide *F_ST_* distribution) and significantly reduced diversity in cultivated cotton (*θ*_π_ ratio < 0.85, corresponding to the bottom 5% left tail of the *θ*_π_ ratio distribution) were defined as domestication selection regions. The thresholds (top 5% for *F_ST_* and bottom 5% for *θ*_π_ ratio) were determined empirically from the genome‐wide distributions following established protocols in domestication genomics. All *F_ST_* and *θ*_π_ ratio values, percentile rankings, and GWAS overlap annotations are available in Table S6.

### 4.16 Map Source

All maps showing geographic distributions were generated using the base map from the Standard Map Service System, Ministry of Natural Resources of China (https://bzdt.ch.mnr.gov.cn), with the review approval number GS(2016)1611 and GS(2016)1585. The original grey fill was converted to boundary lines and the interior was set to blank for improved visualization. No geographic or administrative boundaries were altered.

### 4.17 Statistical Analysis

Phenotypic values were rank‐based inverse normal transformed (RINT) using the Blom method to meet normality assumptions prior to GWAS, as described previously [82]. Data visualization was performed in R (v4.2.0) for bar plots, line graphs, scatter plots, heatmaps, and pie charts, and in Python (v3.8) for 3D Manhattan plots. Data are presented as mean ± SD. Sample sizes (n) are indicated in the figure or figure legends. Two‐sided Student's t‐test was used for two‐group comparisons; Significance levels are denoted as * *P* < 0.05, **P < 0.01, ***P < 0.001, and ****P < 0.0001; ns indicates no significant difference. Pearson correlation was used for i‐Trait correlations.

## Author Contributions

X.G. and J.Z. conceptualized the project. L.W., Y.H., L.Y., Z.H., and Z.S. plant the cotton material. L.W., S.Z., J.P., Y.H., L.Y., Y.Z., M.Z., H.W., Y.Z., S.W., J.H., J.L., X.L., K.N., and Z.Z. performed image collection and conducted the experiments. J.D. performed image recognition and extraction. L.W., T.Z., J.P., performed the bioinformatics analysis. L.W., Y.Z, M.Z., J. L., and X.Z. visualized the graph. H.W. and K.N. created transgenic cotton materials. L.W., J.D., T.Z., T.Z., J. Z. and X.G. prepared the manuscript. All authors read and approved the final manuscript.

## Conflicts of Interest

The authors declare no conflicts of interest.

## Supporting information




**Supporting File 1**: advs76067‐sup‐0001‐SuppMat.docx.

**Supporting File 2**: advs76067‐sup‐0002‐Tables.xlsx.

**Supporting File 3**: advs76067‐sup‐0003‐VideoS1.mov.

**Supporting File 4**: advs76067‐sup‐0004‐VideoS2.mp4.

## Data Availability

The data that support the findings of this study are available from the corresponding author upon reasonable request.
