# Supplementary material for: The Spatiotemporal Genetic Architecture of Seed Vigor in Upland Cotton
Source: Adv Sci (Weinh). 2026 Jun 12:e76067. Online ahead of print. doi: 10.1002/advs.76067 (PMC13336650; doi:10.1002/advs.76067)
Supplement: Supplementary file 1 — Supporting File 1: advs76067‐sup‐0001‐SuppMat.docx. [file ADVS-9999-e76067-s002.docx]

**Supplemental Materials**

**Supplemental Video 1: The working for SeedRanger.**

**Supplemental Video 2: The seed vigor for *FLA2* transgenic cotton.**

**Table S1 Summary of CUCP2 (356 cotton accessions) for high-throughput phenotyping.**

**Table S2 The summary for i-Traits generated in this study.**

**Table S3 GWAS loci information identified for the i-Traits obtained from the high-throughput phenotyping project.**

**Table S4 Gene-based GWAS listed for the i-Trait-associated genes.**

**Table S5 GO analysis for the genes obtained from gene-based GWAS.**

**Table S6 GWAS loci overlapped with sweep selection region for domestication.**

**Table S7 Information for the primers used in this study.**


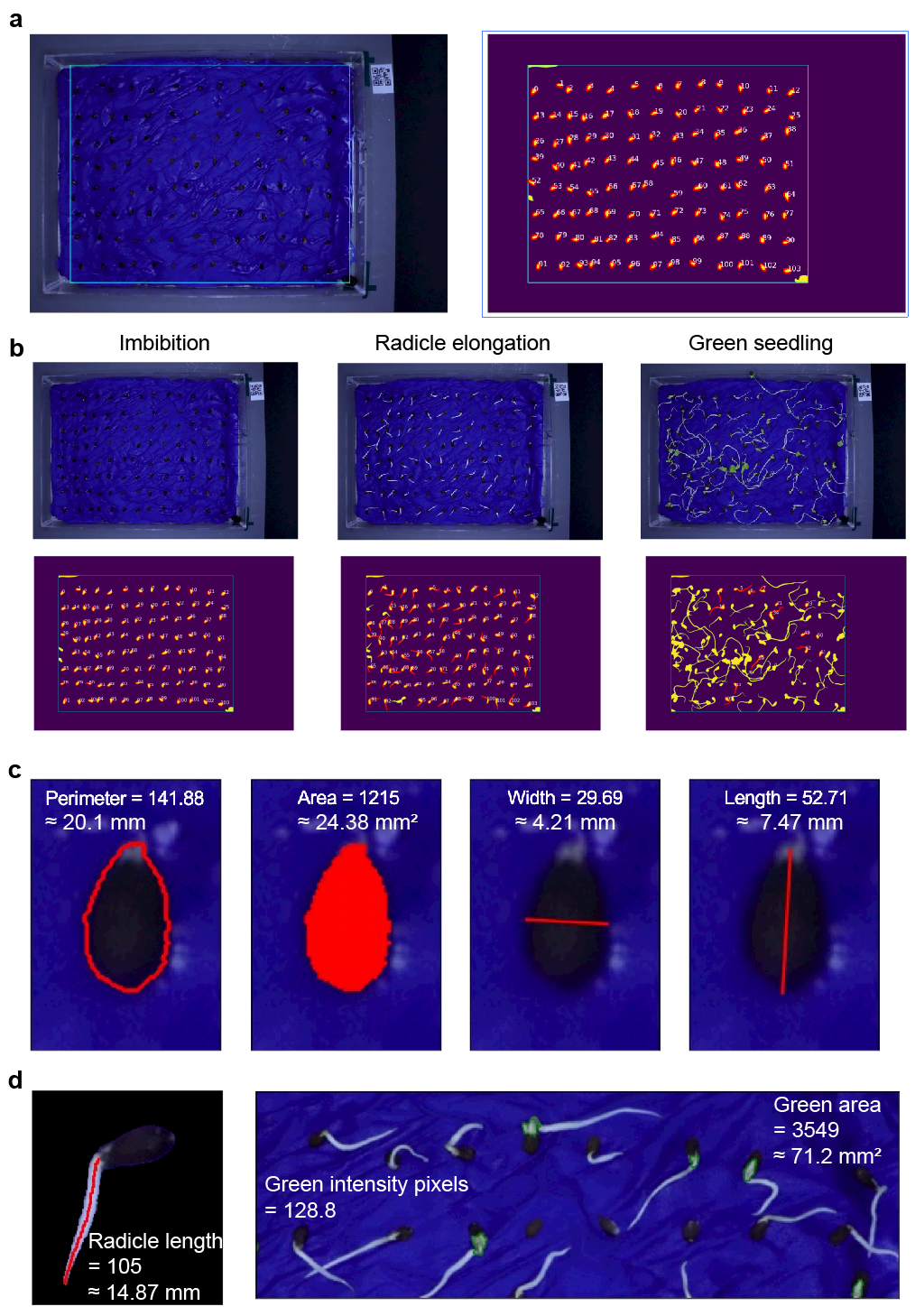


**Fig. S1 | Seed Vigor Phenotypic Trait Extraction**

**a,** the raw photo image captured by SeedRanger (left panel) and the corresponding mask and seed numbers (right panel).

**b,** Representative images showing the original images and corresponding masks and seed numbers at the seed imbibition, germination, and early seedling establishment stages.

**c,** Example of extracting seed perimeter, area, width, and length using a mask developed by computational vision modeling.

**d**, Example of extracting seed radicle length, color value of the green part of the cotyledon, and green area using a mask developed by computational vision modeling.


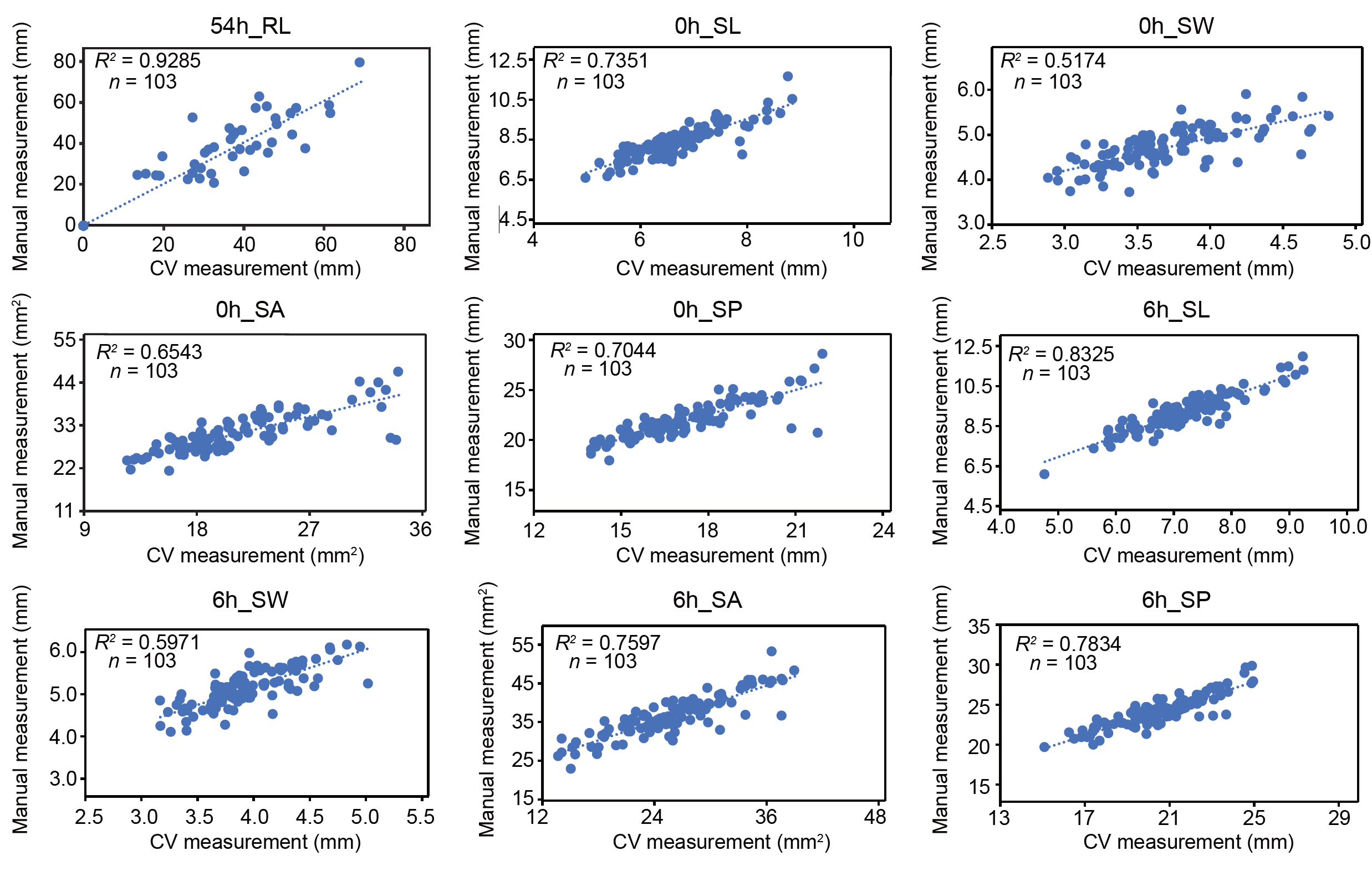
**Fig. S2 | Correlation between manual and automated phenotyping.**

Dot plot comparing seed vigor traits obtained by manual measurement with ImageJ versus automated extraction.

**
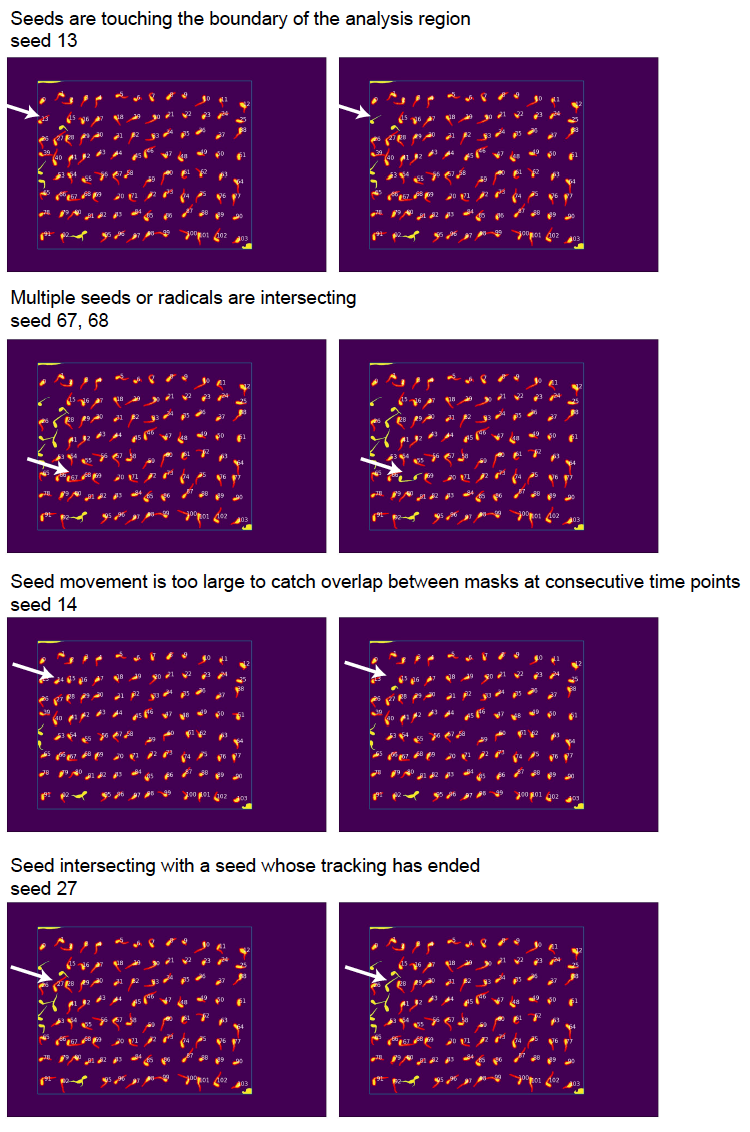
**

**Fig. S3 | Trouble shooting for the failures of i-Trait extraction.**

**
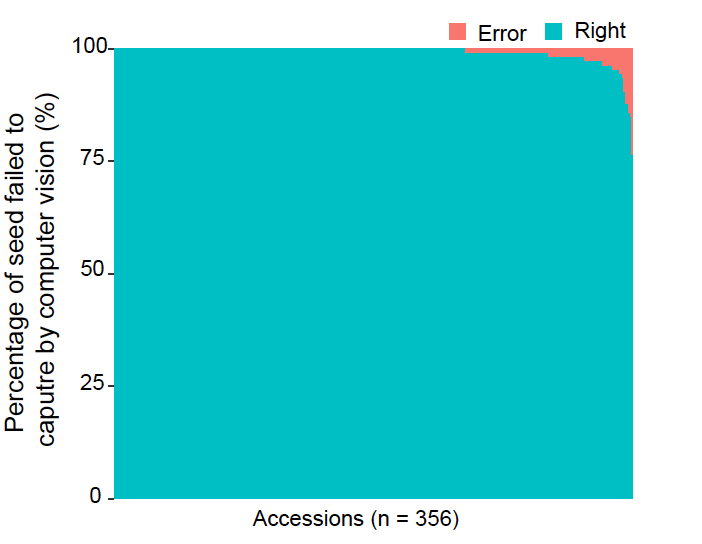
**

**Fig. S4 | The density plot shows the percentage of seed failed to by captured by computational vision.**

**
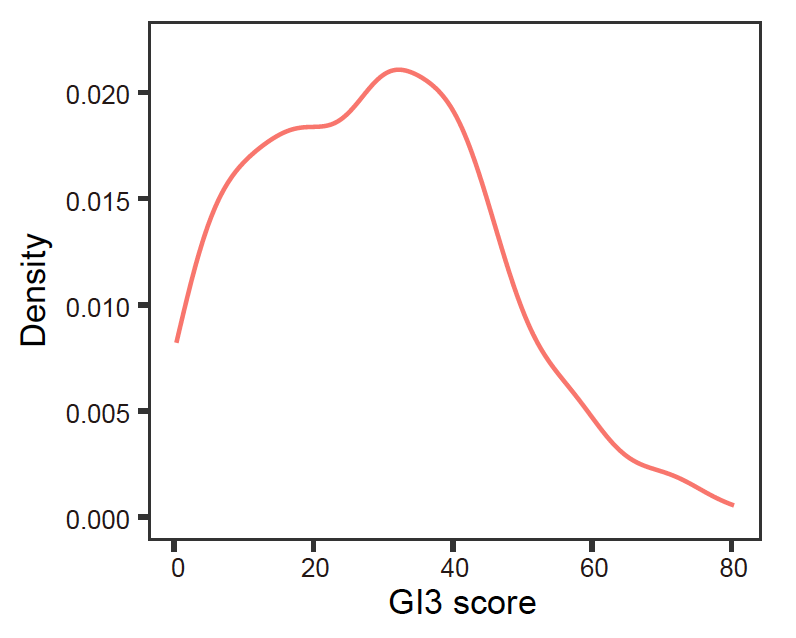
**

**Fig. S5 | The density plot shows the GI in upland cotton population**.


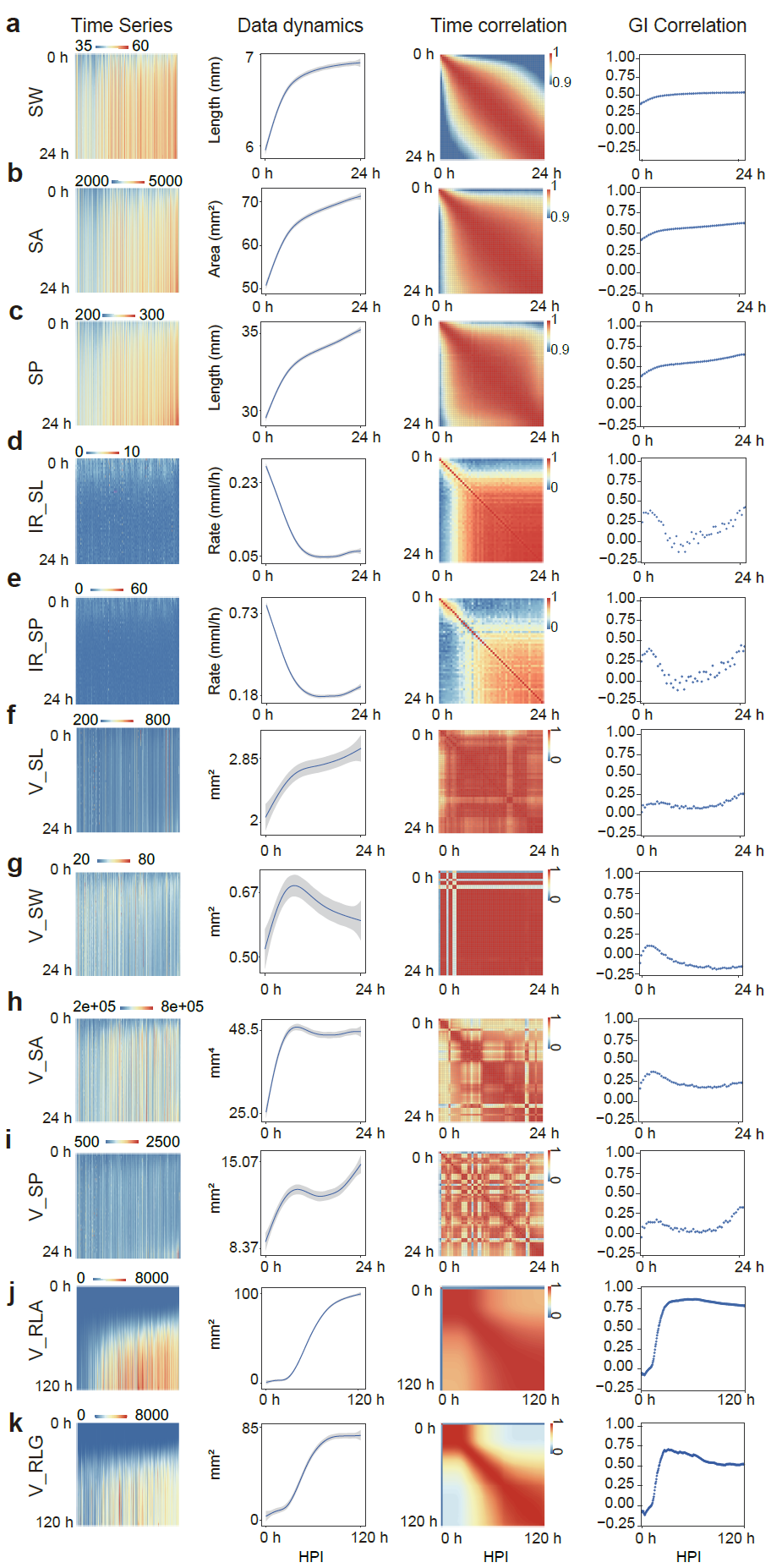


**Fig. S6 | Characterization of i-Traits across developmental stages.**

**a–k,** For each i-Trait, four analytical views are presented: **First column:** Heatmaps showing relative values of (**a**) seed width (SW), (**b**) seed area (SA), (**c**) seed perimeter (SP), (**d**) imbibition rate of SL (IR_SL), (**e**) imbibition rate of SP (IR_SP), (**f**) variance of SL (V_SL), (**g**) variance of SW (V_SW), (**h**) variance of SA (V_SA), (**i**) variance of SP (V_SP) within 24 hours post-imbibition (HPI), and (**j**) variance of radicle length area (V_RLA), (**k**) variance of radicle length growth (V_RLG) within 120 HPI, across 356 accessions sorted by GI3. **Second column:** Line plots showing the mean temporal profile of each corresponding i-Trait. **Third column:** Temporal correlation heatmaps for each i-Trait across all time points. **Fourth column:** Dot plots representing the correlation between each i-Trait time series and GI3.

**
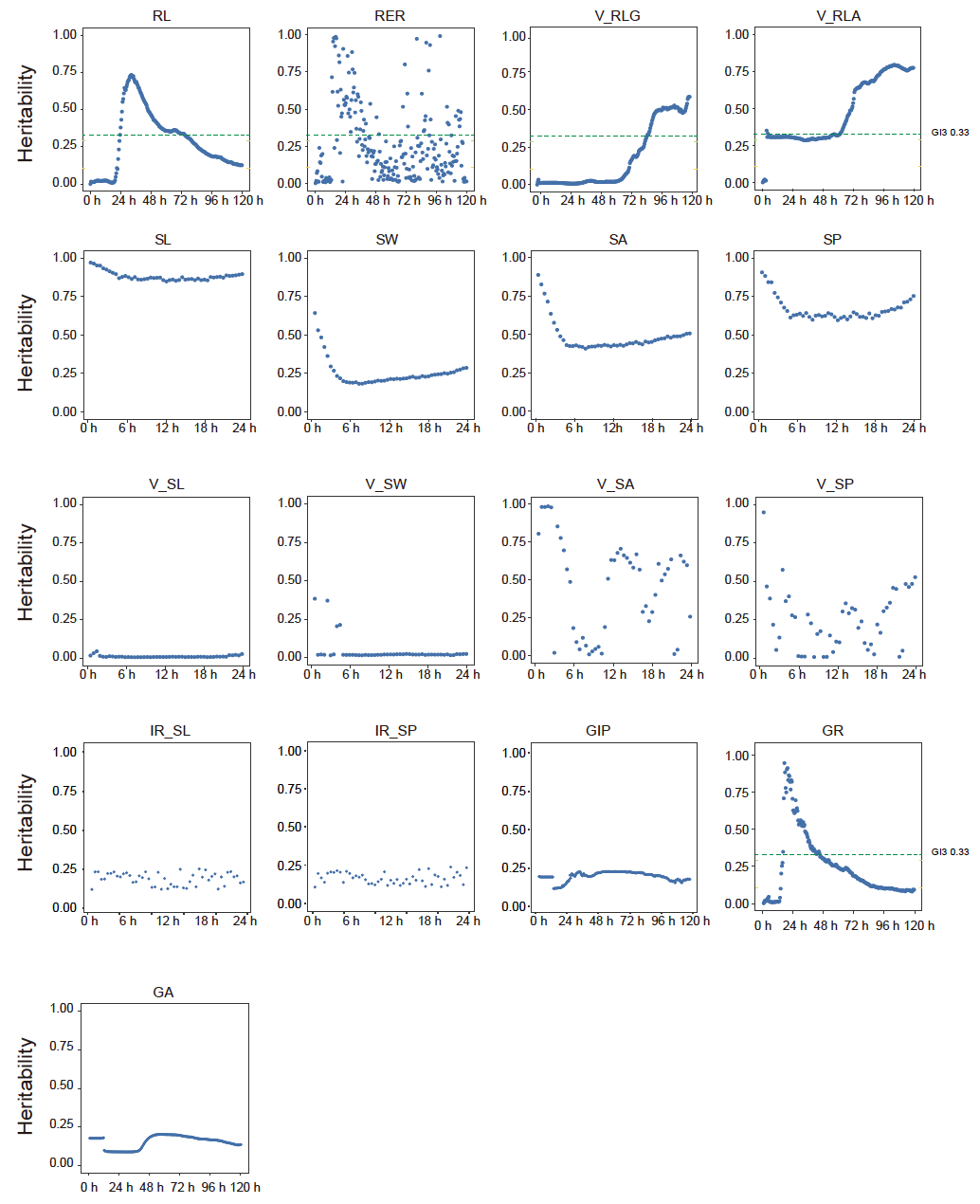
Fig. S7 | The dot plot for heritability of each i-Traits over time series.**


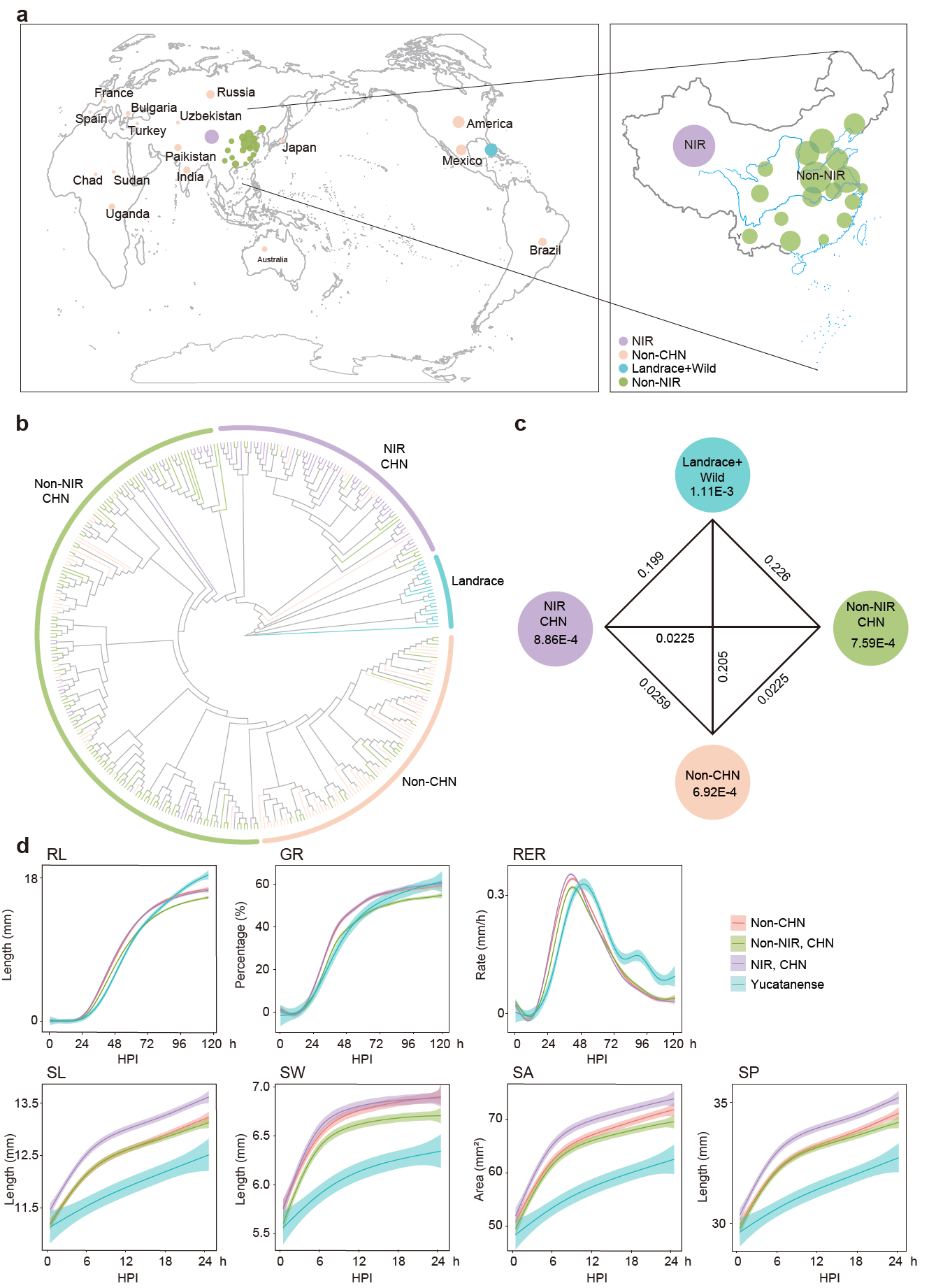


**Fig. S8 | Genomic structure and seed vigor variation in the upland cotton population (CUCP2).**

**a,** Geographic distribution of the collected upland cotton accessions. Base map source: Standard Map Service System, Ministry of Natural Resources of China (https://bzdt.ch.mnr.gov.cn), approval number GS(2016)1611 and GS(2016)1585. The original fill and boundary colors were inverted for visualization purposes; no boundaries were modified.

**b,** Phylogenetic tree constructed from genome-wide SNPs derived from resequencing data.

**c,** Nucleotide diversity (*θ*_π_, within each subgroup) and pairwise population differentiation (*F_ST_*, between subgroups). Circle values represent *θ*_π_ per subgroup; line values indicate *F_ST_*.

**d,** Temporal phenotypic profiles of seven i-Traits—radicle length (RL), germination rate (GR), radicle emergence rate (RER), seed length (SL), seed width (SW), seed area (SA), and seed perimeter (SP)—across four genetic subgroups. Traces show mean values per time point over the germination period.


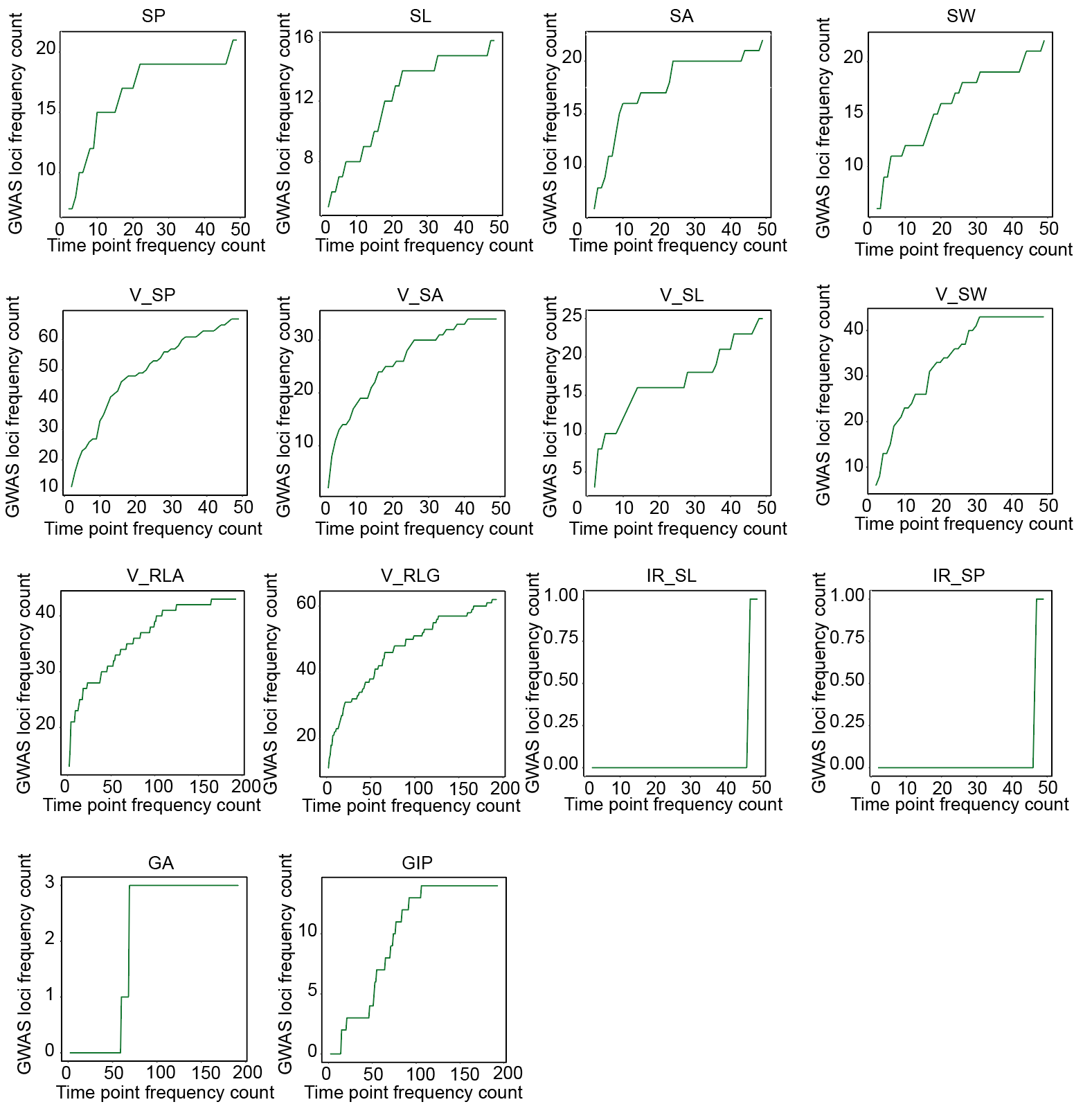
**Fig. S9 | Efficiency of GWAS locus detection across time points. The line plot shows the number of significant loci detected as a function of the number of time points included in the analysis.**


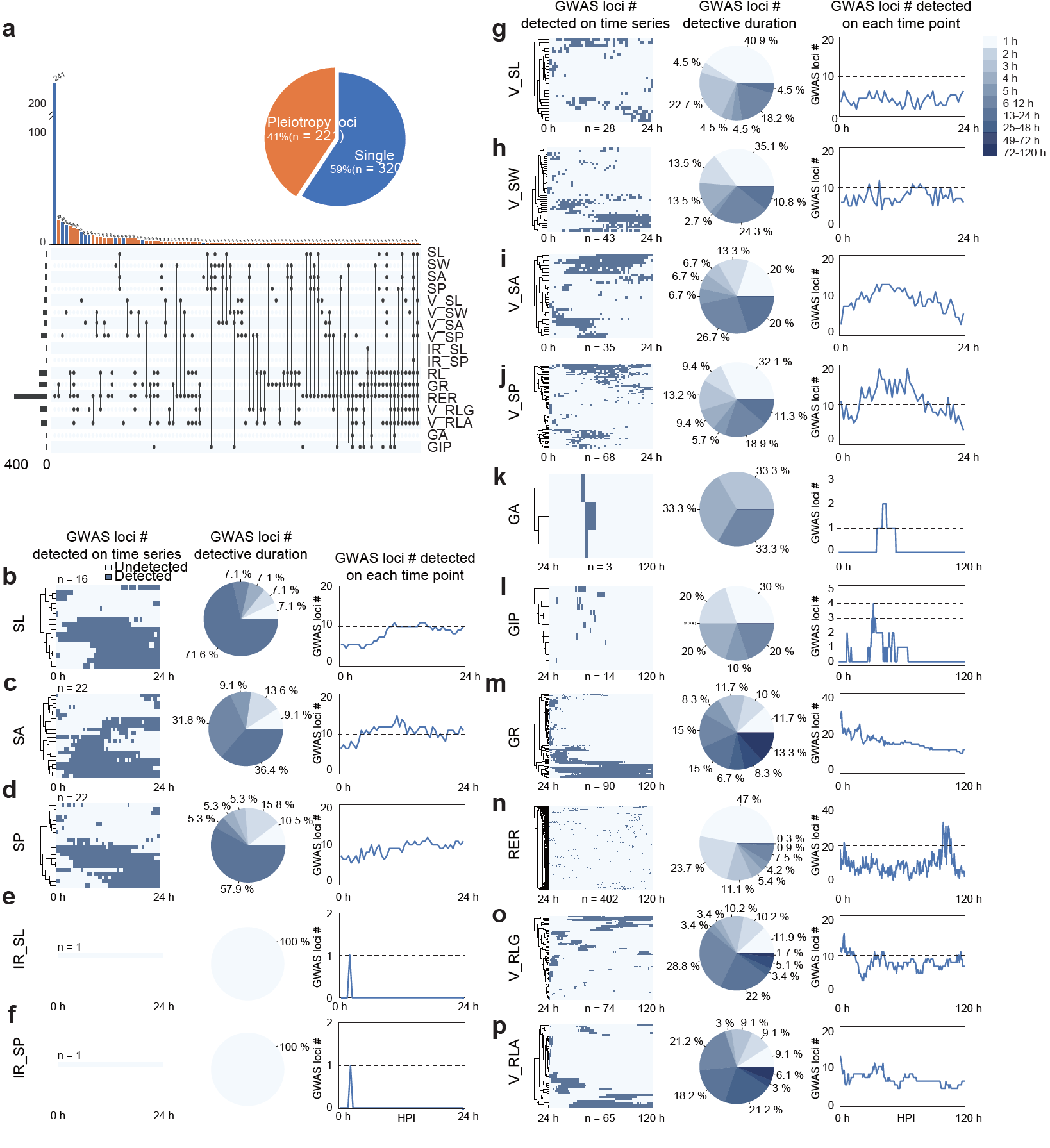
**Fig. S10 | Dynamic patterns of quantitative trait loci (QTLs) governing seed vigor.**

**a,** Left: UpsetR plot illustrating the number of QTLs identified for each i-Trait category. Right: Proportion of pleiotropic loci (associated with multiple i-Traits) versus single-effect loci.

**b–p**, For each i-Trait—seed length (SL), seed area (SA), seed perimeter (SP), imbibition rate of SL (IR_SL), imbibition rate of SP (IR_SP), variance of SL (V_SL), variance of SW (V_SW), variance of SA (V_SA), variance of SP (V_SP), green area (GA), green intensity pixels (GIP), germination rate (GR), radicle emergence rate (RER), variance of radicle length growth (V_RLG), and variance of radicle length area (V_RLA)—three analytical views are provided: **First column**: Temporal detection matrix. Dark blue indicates a significant GWAS locus was detected at a given time point; light blue indicates no significant association. **Second column**: Pie chart showing the proportion of QTLs with an effective duration of 1–24 hours versus 1–120 hours. **Third column**: Time-course profile of the number of significant GWAS loci detected at each time point.


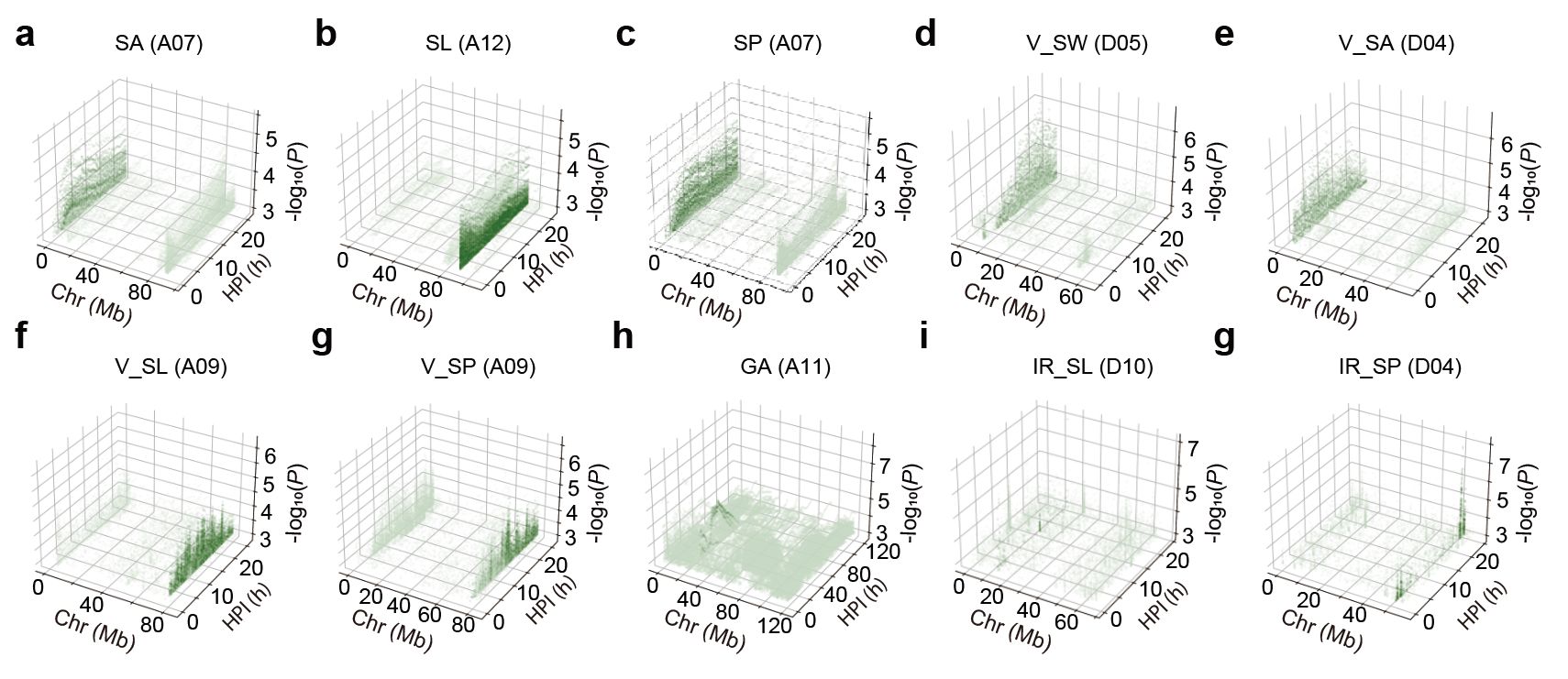


**Fig. S11 | Identification of persistent genetic loci underlying seed vigor.** Visualization of i-Trait-associated loci via 3D Manhattan plots, highlighting those with sustained significance (*P* ＜ 3.89 × 10⁻⁶; dark green) across the germination time course.


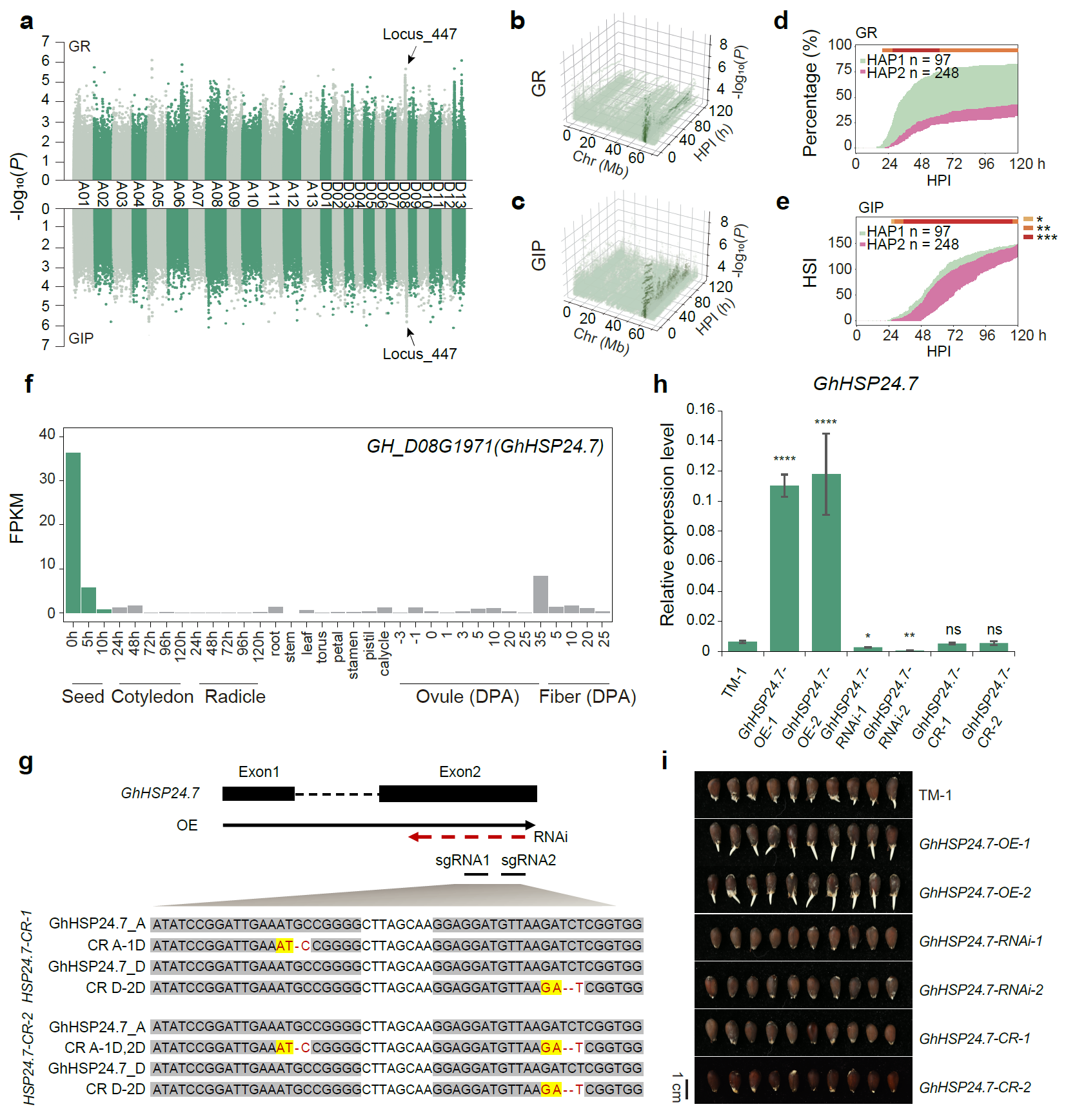


**Fig. S12 | Functional characterization of Locus_447 and its candidate gene *GhHSP24.7* in germination-related traits.**

**a,** Manhattan plot of Locus_447 associated with germination rate (GR) and green intensity pixels (GIP).

**b, c,** Three-dimensional Manhattan plots of Locus_447 for GR (**b**) and GIP (**c**) across time.

**d, e,** Phenotypic variation of GR (**d**) and GIP (**e**) over 120 h in accessions grouped by haplotype (Hap1/Hap2) of Locus_447. **P* < 0.05, ***P* < 0.01, ****P* < 0.001, by two-sided Student’s *t*-test.

**f,** *GhHSP24.7* expression (FPKM) across different cotton tissues.

**g,** Schematic of *GhHSP24.7* overexpression (OE), RNAi, and CRISPR-Cas9 editing constructs; lower panel shows sequencing results of the *GhHSP24.7-CR* line.

**h,** Relative expression of GhHSP24.7 in OE, RNAi, and CR lines. Data are mean ± s.d.; **P* < 0.05, ***P* < 0.01, *****P* < 0.0001 by two-sided Student’s *t*-test; n ≥ 3 biologically independent samples.

**i,** Germination phenotypes of *GhHSP24.7-OE*, *GhHSP24.7-RNAi*, and *GhHSP24.7-CR* seeds at 36 h post-imbibition.

**Note:** The genetic intervals of Locus_447 for multiple traits colocalized to a shared genomic region. Haplotype analysis was therefore performed using the lead SNP from each trait: Germination rate (GR; D08:60924087) and green intensity pixel (GIP; A07:60924074).


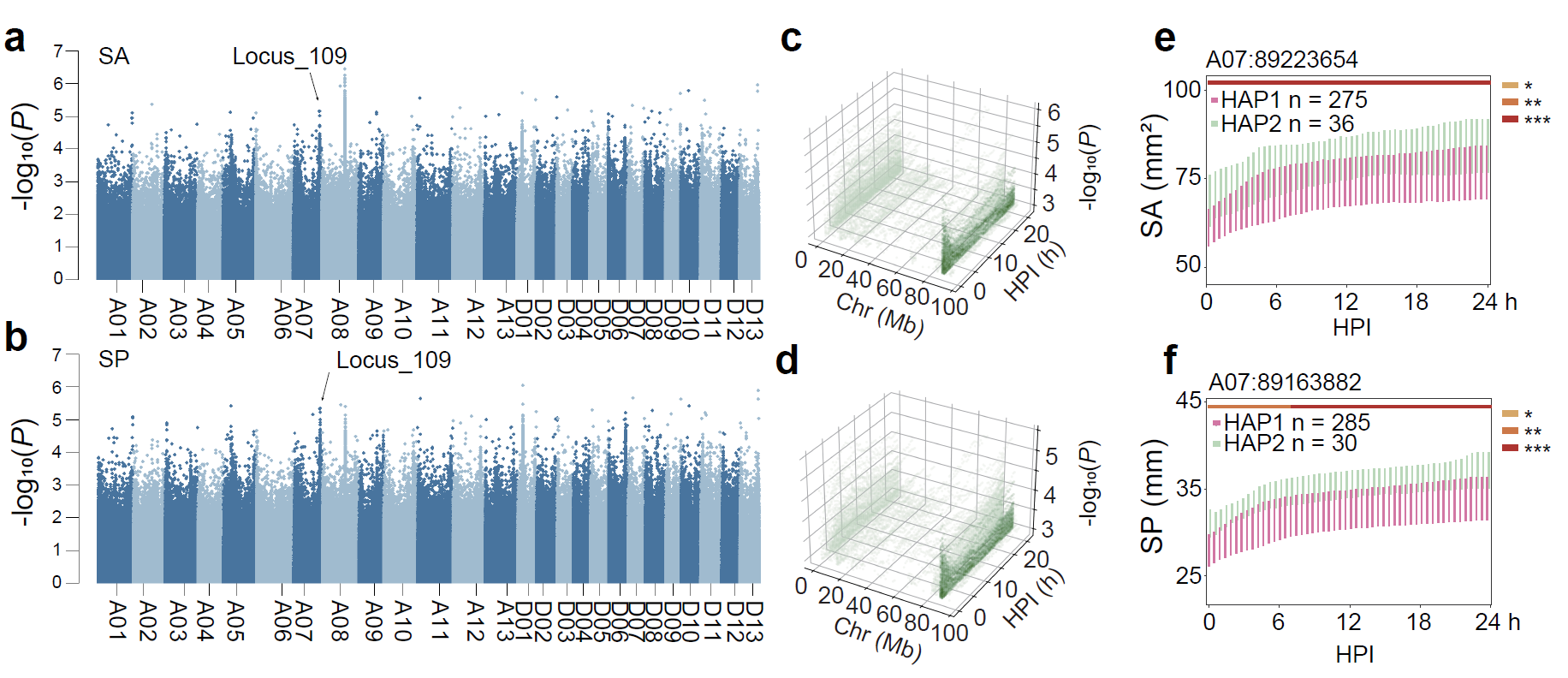


**Fig. S13 | Functional characterization of Locus_109 for seed area and seed perimeter traits.**

**a, b,** Manhattan plots of Locus 109 for seed area (SA; **a**) and seed perimeter (SP; **b**).

**c, d,** Three-dimensional Manhattan plots showing dynamic associations of Locus_109 with SA (**c**) and SP (**d**) over time.

**e, f,** Phenotypic variation of SA (**e**) and SP (**f**) over 120 h in accessions grouped by haplotype (Hap1/Hap2) defined by the lead SNPs at Locus_109.

**Note:** The genetic intervals of Locus_109 for multiple traits colocalized to a shared genomic region. Haplotype analysis was therefore performed using the lead SNP from each trait: seed Area (SA; A07: 89223654) and Seed Perimeter (SP; A07: 89163882). **P* < 0.05, ***P* < 0.01, ****P* < 0.001, by two-sided Student’s *t-*test.

**
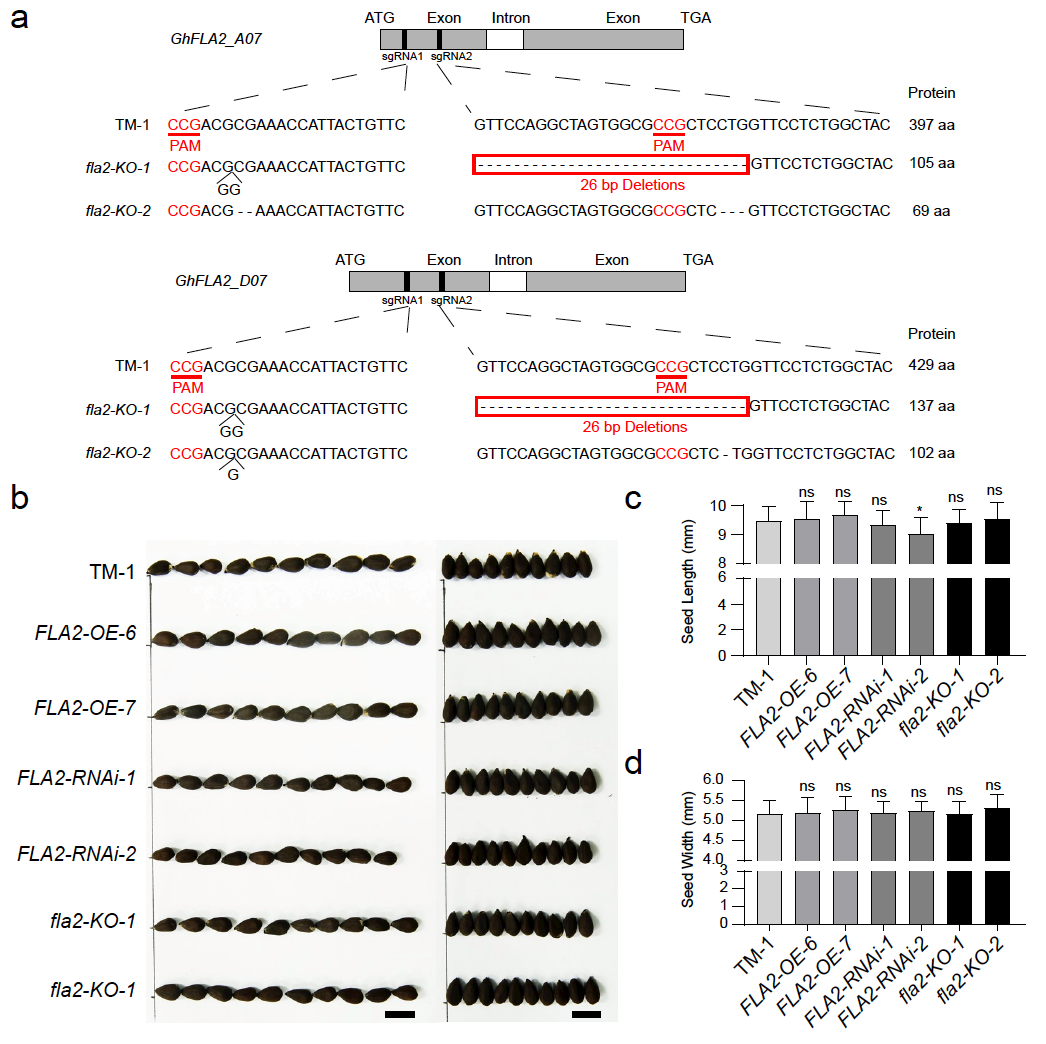
Fig. S14 | *FLA2* regulates seed size based on transgenic validation.**

**a,** Sequencing results of the targeted region in the *fla2* knockout (*fla2-KO*) lines.

**b,** Representative images showing seed size variation among wild-type and *FLA2* transgenic lines.

**c,** Quantification of seed length (**c**) and seed width (**d**) across genotypes. Data are presented as mean ± s.d.; **P*< 0.05 by two-sided Student’s *t*-test; n ≥ 3 biologically independent samples.


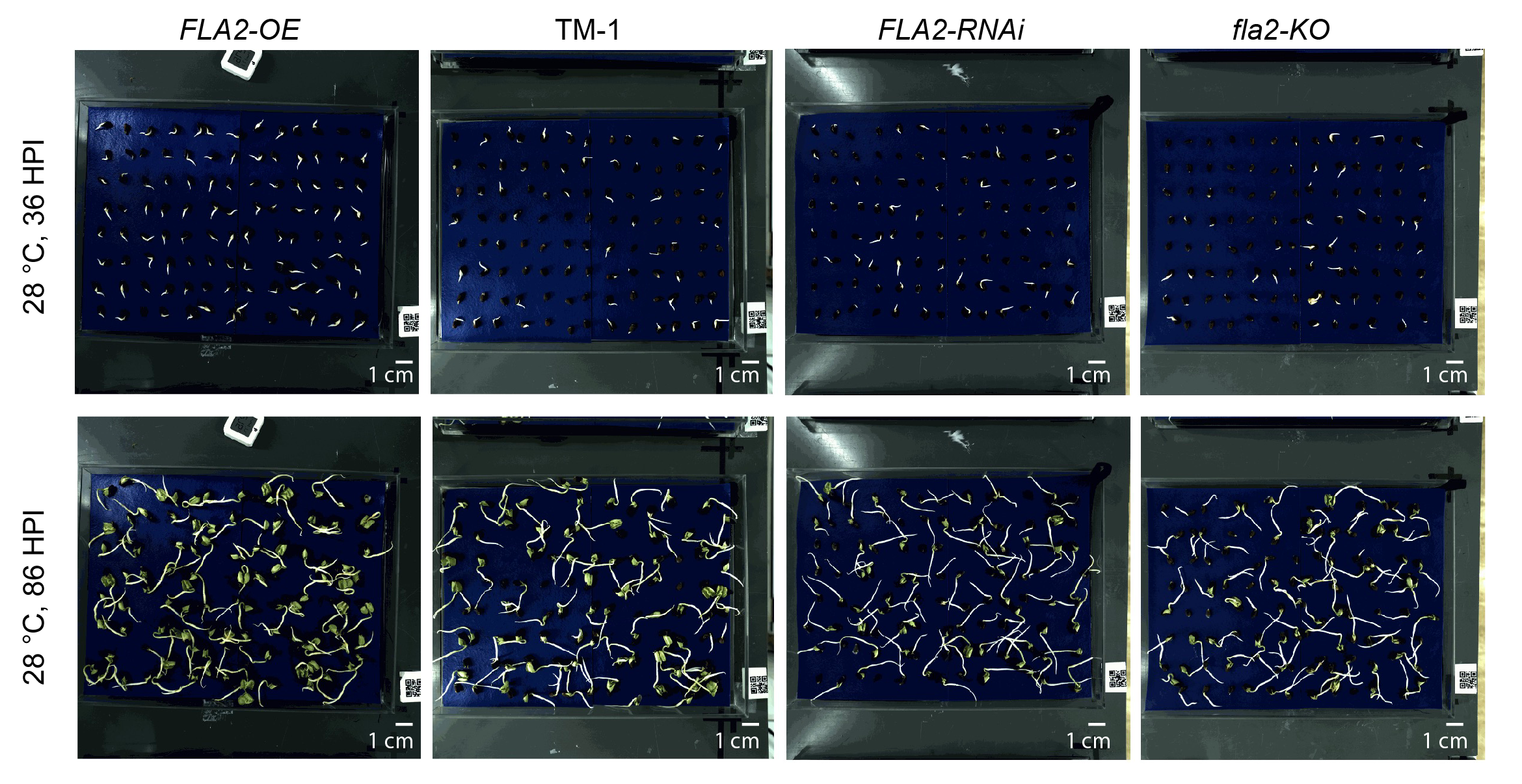


**Fig. S15 | *FLA2* modulates seed germination vigor.**

Visual phenotypes of germinating seeds from *FLA2* overexpression (*FLA2-OE*), wild-type (TM-1), RNA interference (*FLA2-RNAi*), and knockout (*fla2-KO*) lines at 36 hours post-imbibition (36 HPI, top row) and 86 HPI (bottom row) under standard conditions (28°C). Scale bar, 1 cm.


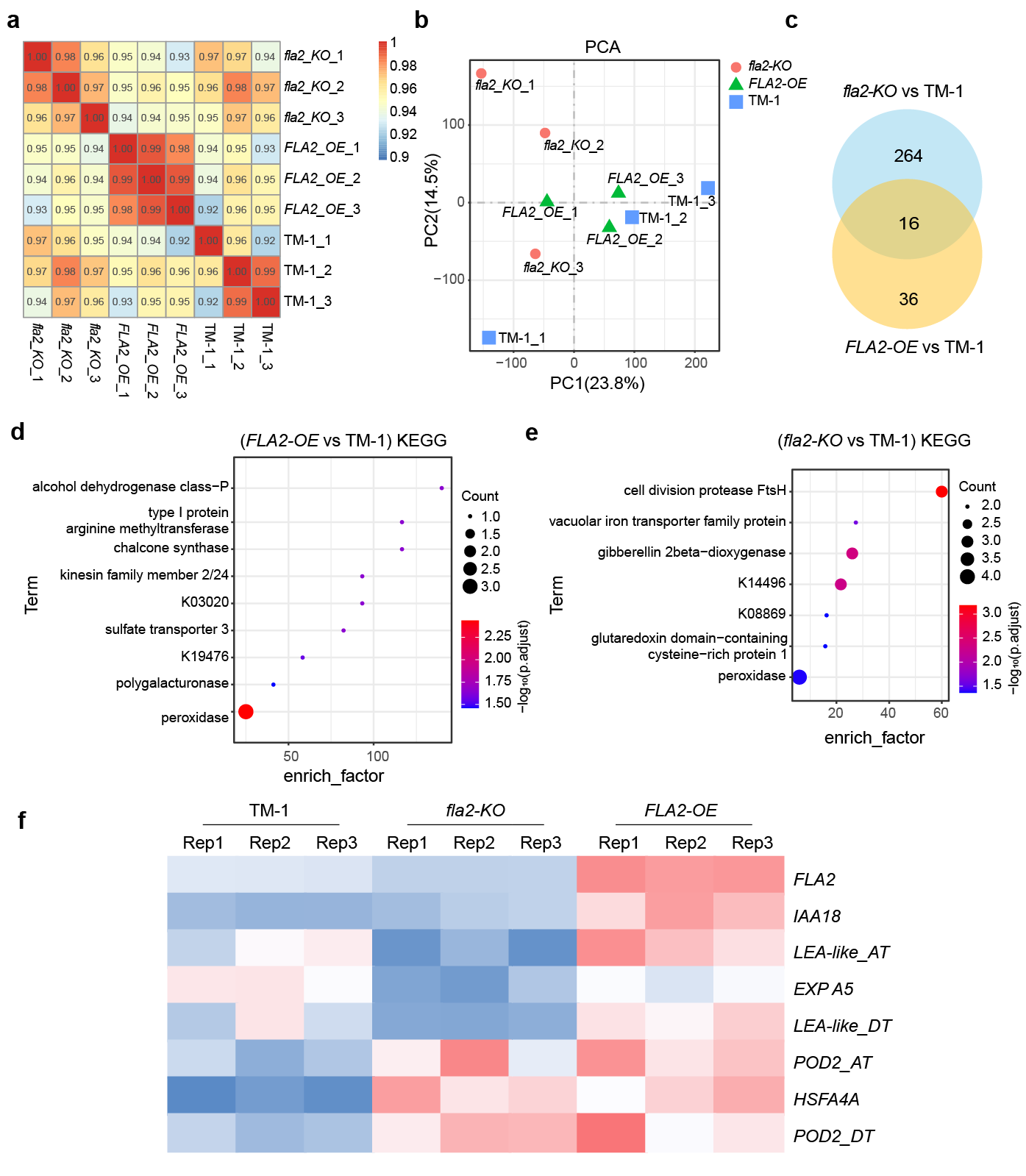


**Fig. S16 | Transcriptome profiling of *FLA2* transgenic lines.**

**a,** Pearson correlation heatmap of RNA-seq samples from the three biological replicates of TM-1, *FLA2-OE*, and *fla2-KO* lines.

**b,** PCA plot showing clustering of transcriptome profiles by genotype.

**c,** Venn diagram showing overlap of differentially expressed genes (DEGs) between *FLA2-OE* vs *TM-1* and *fla2-KO* vs *TM-1* comparisons*.*

**d, e,** KEGG pathway enrichment analysis of DEGs in *FLA2-OE* (**d**) and *fla2-KO* (**e**). Dot size represents gene count; color indicates adjusted *P*-value.

**f,** Heatmap of FPKM of *FLA2* and putative downstream genes, including IAA18, LEA-like, EXP A5, POD2, and HSFA4A, across biological replicates.


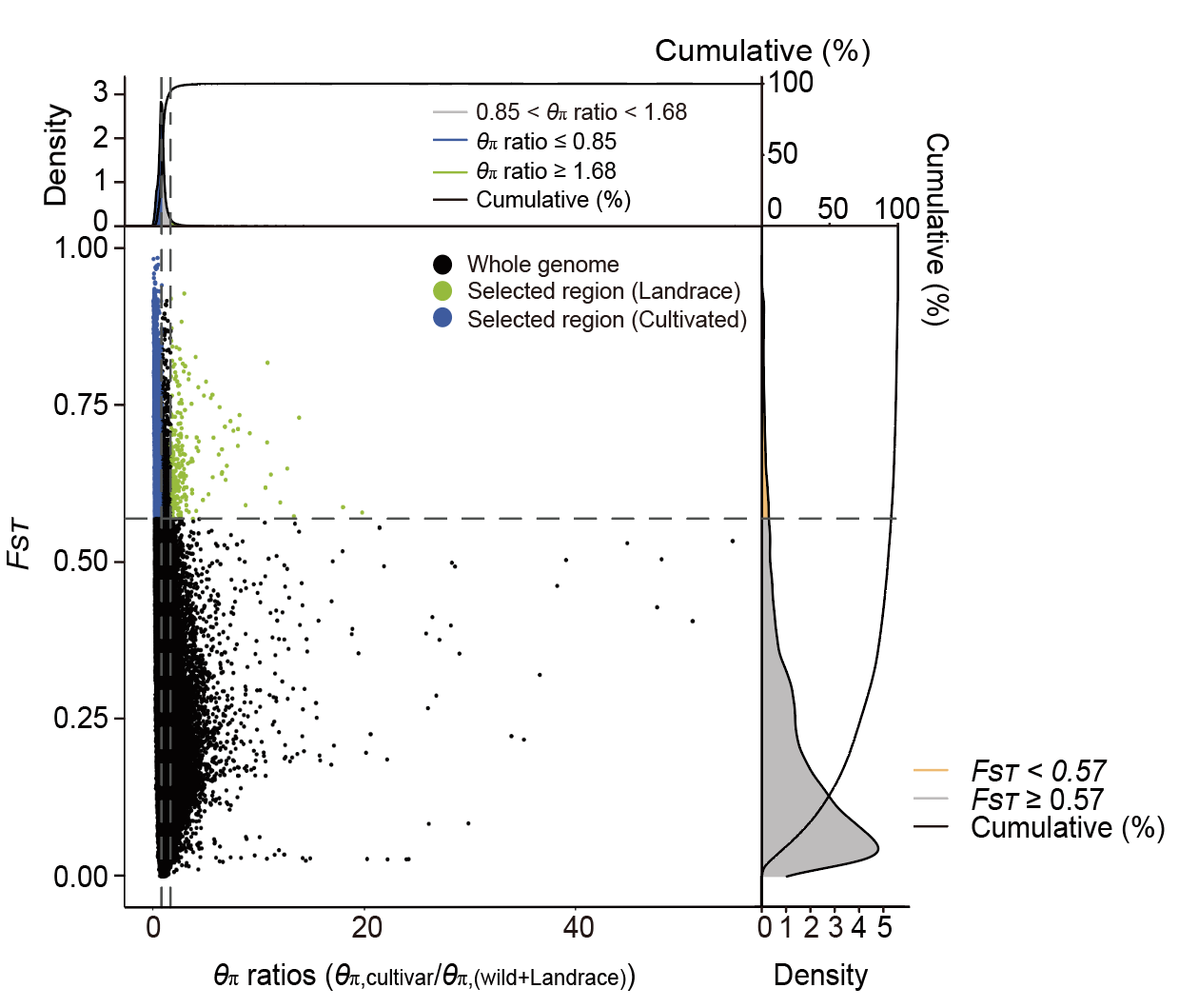


**Fig. S17 | Genome-wide landscape of domestication selection in the upland cotton**

Joint distribution of population differentiation (*F_ST_*) and nucleotide diversity ratio (*θ*_π_) across the genome. The *x*-axis shows *θ*_π_ ratios (*θ*_π, cultivar_/*θ*_π, (wild+Landrace)_), and the *y*-axis shows *F_ST_* values between cultivar and wild/landrace cotton. Each dot represents a 100-kb genomic window. Black dots indicate genome-wide background; blue dots indicate regions under selection in cultivated cotton (domestication sweeps: *F_ST_* ≥ 0.57 and *θ*_π_ ratio ≤ 0.85); green dots indicate regions under selection in landraces. The dashed horizontal line marks the *F_ST_* threshold (0.57, top 5%); the vertical dashed line marks the *θ*_π_ ratio threshold (0.85, bottom 5%). The upper and right panels show marginal density distributions of *F_ST_* and *θ*_π_ ratios, respectively, with cumulative percentages indicated. Regions simultaneously exhibiting high *F_ST_* (≥ 0.57) and low *θ*_π_ ratio (≤ 0.85) represent domestication sweeps with strong selection signatures.


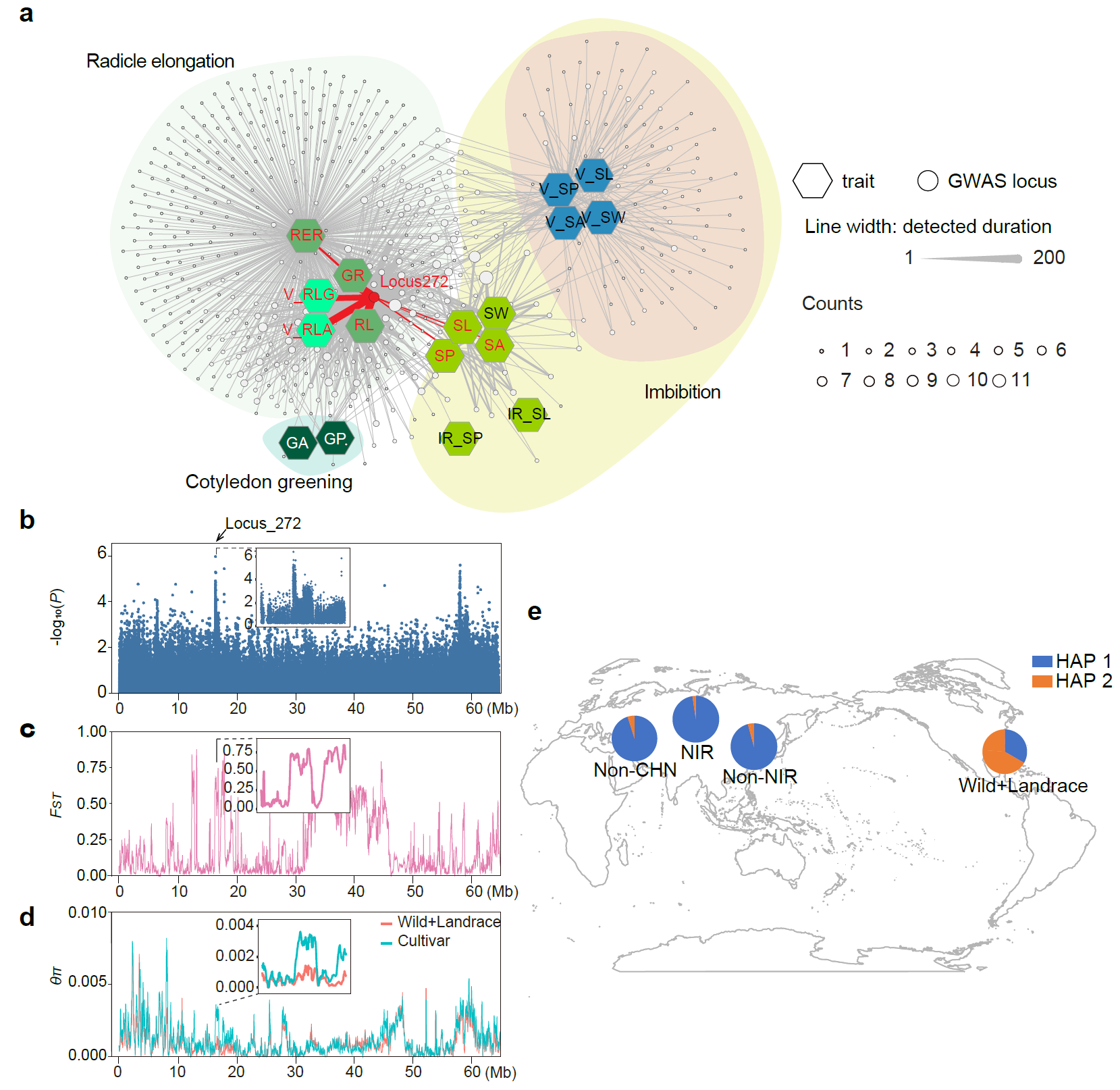


**Fig. S18 | Locus_272 contributes to seed vigor and shows signatures of selection.**

**a,** Network diagram showing the pleiotropic effects of Locus_272 (center, red) on seed vigor i-Traits (colored hexagons) across three developmental stages: Imbibition (yellow background), Radicle elongation (green), and Cotyledon greening (teal). Small circles represent individual GWAS loci; line width indicates detection duration (1-200 time points); circle size represents allele counts (1-11). **b,** Manhattan plot showing the association signal of Locus_272 on chromosome D01 (*x*-axis: genomic position in Mb; *y*-axis: -log₁₀(*P*-value)). Inset shows zoomed view of the peak region.

**c,** Genome-wide distribution of fixation index (*F_ST_*) between cultivated and (wild+landrace) cotton across chromosome D01. The inset highlights elevated *F_ST_* values at Locus_272 (boxed region), indicating strong population differentiation.

**d,** Nucleotide diversity (*θ*_π_) across chromosome D01 comparing (Wild+Landrace) (red) and Cultivar (cyan). The inset shows markedly reduced nucleotide diversity in cultivars at Locus_272, consistent with a selective sweep signature.

**e,** Geographical distribution of Locus_272 haplotypes (HAP1: blue; HAP2: orange) across cotton populations: NIR, Non-NIR, Non-CHN, and (Wild+Landrace). Base map source: Standard Map Service System, Ministry of Natural Resources of China (https://bzdt.ch.mnr.gov.cn), approval number GS(2016)1611. The original fill and boundary colors were inverted for visualization purposes; no boundaries were modified.
